# Supplementary material for: Reasons for non-adherence to cardiometabolic medications, and acceptability of an interactive voice response intervention in patients with hypertension and type 2 diabetes in primary care: a qualitative study
Source: BMJ Open. 2017 Aug 11;7(8):e015597. doi: 10.1136/bmjopen-2016-015597 (PMC5724082; doi:10.1136/bmjopen-2016-015597)
Supplement: supplementary file 1 [file bmjopen-2016-015597supp001.pdf]

Draft interview schedule for HCPs: "Supporting medication adherence in people with long-term conditions"

FACE-TO-FACE INTERVIEWS WITH HEALTH CARE PROVIDERS

DRAFT interview schedule HCPs (v5)

Introduction

- introduce myself
- give an overview of the aims of the interview
- consent issues
    - ask if participants have read the information sheet and if they have any questions
        - if *not*, overview the information included
    - clarify that they can withdraw from the interview at any time, without giving a reason
    - clarify that the interview will be recorded, explain the use of transcripts and the confidentiality issues
- obtain signed consent

Draft interview schedule for HCPs: "Supporting medication adherence in people with long-term conditions"

Experience with medication adherence

- How many patients have you seen with long term conditions?
- How often do you discuss medication adherence with patients with long term conditions? / How many years have you been addressing mediation adherence?
- How many patients have you seen with diabetes, hypertension or both?
- How often do you discuss medication adherence with patients with diabetes and/or hypertension?

Views on medication adherence and factors that influence medication adherence

- Do people with diabetes and hypertension take all their medications as prescribed? - if no, what percentage of participants do not take all their medications as prescribed?
    - Is there any difference between people with diabetes and people with hypertension? - If yes, how do they differ?/ If no, why do you think so? (*use debriefing questions and prompts*)

Views on factors that influence medication adherence

- What reasons do people with diabetes and/or hypertension report for taking their medications as prescribed?
- What benefits do people with diabetes and hypertension report that they gain from taking their medications as prescribed?
- What people expect from taking their medications?
- What sort of things do people ask you about taking their medications?

DRAFT interview schedule HCPS (v5)

Draft interview schedule for HCPs: "Supporting medication adherence in people with long-term conditions"

- What is your view about how people with diabetes and/or hypertension take their medications?
- What reasons do people report for not taking all their medications as prescribed?

Current practices to address medication adherence and recommendations for improvement

- What do you usually say to motivate people with diabetes and/or hypertension to take their medications regularly as prescribed?
- What do you usually say to people with diabetes and/or hypertension to support them to take their medications as prescribed?
- What do you usually do to support people with diabetes and/or hypertension to take their medications as prescribed?
- How do you see these practices? how do they support people to take their medications as prescribed?
- When do you think people with long term conditions need support more? - how this could be done?
- Do you think a text or voice message intervention could be helpful to support people with diabetes/hypertension to take their medications ? - if yes, how? if no, why?
- What kind of support do you think an text or voice message intervention could provide?/ What kind of information do you think a text or voice message intervention could provide? Can you give me an example?

DRAFT interview schedule HCPs (v5)

Draft interview schedule for HCPs: "Supporting medication adherence in people with long-term conditions"

- How often do you think a intervention message could be helpful for people with diabetes and hypertension?
- Do you think a text or voice message intervention could be usefully integrated in the primary care?/ if yes, how do you think such intervention could be integrated in the primary care?/ if no, why do you think so? any recommendations for current practices?

DRAFT interview schedule HCPs (v5)

Draft interview schedule for HCPs: "Supporting medication adherence in people with long-term conditions"

Debriefing questions

- Is there anything else you would like to add about medication adherence?
- Are there any questions about the procedure generally?

Interview prompts

Identifying thoughts/ views:

- Could you please tell me a bit more about that?
- Could you please give me an example of ....?
- Anything else?

Identifying meanings:

- Please correct me if I' m wrong, you said that (rephrase what the interviewee said)...do you have any ideas how that might work?
- Why do you think this is important to you/ to people with long term conditions?
- What do you mean by....?
- What does this depends on?

DRAFT interview schedule HCPs (v5)
