# Supplementary material for: Reasons for non-adherence to cardiometabolic medications, and acceptability of an interactive voice response intervention in patients with hypertension and type 2 diabetes in primary care: a qualitative study
Source: BMJ Open. 2017 Aug 11;7(8):e015597. doi: 10.1136/bmjopen-2016-015597 (PMC5724082; doi:10.1136/bmjopen-2016-015597)
Supplement: supplementary file 2 [file bmjopen-2016-015597supp002.pdf]

FACE-TO-FACE INTERVIEWS WITH PEOPLE WITH LONG TERM CONDITIONS

Introduction

- introduce myself
- give an overview of the aims of the interview
- consent issues
    - ask if participants have read the information sheet and if they have any questions
        - if *not*, overview the information included
    - clarify that they can withdraw from the interview at any time, without giving a reason
    - clarify that the interview will be recorded, explain the use of transcripts and the confidentiality issues
- obtain signed consent

DRAFT interview schedule pLTCs(v4)

DRAFT interview schedule for pLTCs: "Supporting medication adherence in people with long-term conditions"

First interview

Assessment of the factors that influence medication adherence and current medication taking

- How many medications have you been prescribed?
- Could you describe to me what your medications are?
- What is/are the reason(s) you take your medications?
- What do you expect by taking your medications?
- How do you take your medications?
- When do you take your medications?
- Where do you usually take your medications?
- What do you usually do when you take your medications?
- What helps you to take your medications regularly? / What do you do to help you take your medications?
- Do you take all these medications? / How many of these medications do you take?
  - If yes:
    - what helps you take all your medications as prescribed?
  - If not:
    - which of these do you take and which you do not take?
    - what might be the reasons?
    - what do you think would make it easier to take all your medications as prescribed?
    - what could help you address these reasons?
    - how do you think this could be done?

DRAFT Interview schedule pLTCs(v4)

- what kind of information/support would you need? / could you give me an example?
- do you think a text or voice message intervention could help you to achieve this?

Debriefing questions

- Is there anything else you would like to add about medication adherence?
- Are there any questions about the procedure generally?

Interview prompts

Identifying thoughts/ views:

- Could you please tell me a bit more about that?
- Could you please give me an example of ....?
- Anything else?

Identifying meanings:

- Please correct me if I' m wrong, you said that (rephrase what the interviewee said)...do you have any ideas how that might work?
- Why do you think this is important to you/ to people with long term conditions?
- What do you mean by....?
- What does this depends on?

DRAFT interview schedule pLTCs(v4)

Second interview

Instructions to participants

We will shortly be beginning a study to support people with long-term conditions to take their medications as prescribed.

For this study, we have developed some intervention materials about people's reasons in taking or not taking their medications. We want to check that people understand the intervention materials in the way that we meant them. To do this, I am going to ask you to 'think-aloud' as you go through these materials. So I want you to tell me everything that you are thinking as you read/hear all the information and decide how to answer it. I would like you to talk constantly. I do not want you to plan out what you say or try to explain to me what you are saying. Just act as if you are alone in the room. If you are silent for any long period of time, I will ask you to 'keep talking' or I will prompt you with some questions.

DRAFT interview schedule pLTCs v(4)
