# Supplementary material for: Reasons for non-adherence to cardiometabolic medications, and acceptability of an interactive voice response intervention in patients with hypertension and type 2 diabetes in primary care: a qualitative study
Source: BMJ Open. 2017 Aug 11;7(8):e015597. doi: 10.1136/bmjopen-2016-015597 (PMC5724082; doi:10.1136/bmjopen-2016-015597)
Supplement: Author's manuscript [file bmjopen-2016-015597.draft_revisions.pdf]

BMJ Open is committed to open peer review. As part of this commitment we make the peer review history of every article we publish publicly available.

When an article is published we post the peer reviewers' comments and the authors' responses online. We also post the versions of the paper that were used during peer review. These are the versions that the peer review comments apply to.

The versions of the paper that follow are the versions that were submitted during the peer review process. They are not the versions of record or the final published versions. They should not be cited or distributed as the published version of this manuscript.

BMJ Open is an open access journal and the full, final, typeset and author-corrected version of record of the manuscript is available on our site with no access controls, subscription charges or pay-per-view fees (http://bmjopen.bmj.com).

If you have any questions on BMJ Open's open peer review process please email editorial.bmjopen@bmj.com

BMJ Open

Reasons for non-adherence to medication and acceptability of an interactive voice response intervention in patients with hypertension and type 2 diabetes in primary care. A qualitative study

Journal:	*BMJ Open*
Manuscript ID	bmjopen-2016-015597
Article Type:	Research
Date Submitted by the Author:	20-Dec-2016
Complete List of Authors:	Kassavou, Katerina; University of Cambridge, Public Health and Primary Care ; University of Cambridge,<br>Sutton, Stephen; University of Cambridge,
**Primary Subject Heading**:	Cardiovascular medicine
Secondary Subject Heading:	Communication, Diabetes and endocrinology, Health policy, Health services research, Medical education and training
Keywords:	Medication adherence, interactive voice response, Hypertension < CARDIOLOGY, Telemedicine < BIOTECHNOLOGY & BIOINFORMATICS, DIABETES & ENDOCRINOLOGY

SCHOLARONE™
Manuscripts

For peer review only

Title: Reasons for non-adherence to medication and acceptability of an interactive voice response intervention in patients with hypertension and type 2 diabetes in primary care. A qualitative study

Author and co-author name: Katerina Kassavou and Stephen Sutton

Name, postal address, email, telephone and fax number of the corresponding author: Katerina Kassavou (BSc in Psychology, MSc in Clinical Health Psychology, PhD in Health Psychology). Research Associate, Behavioural Science Group, University of Cambridge, School of Clinical Medicine, Institute of Public Health and Primary Care, The Primary Care Unit, Forvie Site, Robinson Way, Cambridge, CB2 0SR. kk532@medschl.cam.ac.uk. +44(0)1223330456

The full names, institutions, city, and country of all co-authors: Stephen Sutton, (BSc in Combined Studies, Psychology main, MSc in Social Psychology, MSc in Computer Science, PhD in Social Psychology). Professor in Behavioural Science, University of Cambridge, Cambridge, UK.

Up to five keywords or phrases suitable for use in an index: Medication adherence, interactive voice response, hypertension, type 2 diabetes.

Word count: 4,766 (excluding title page, abstract, references, figures and tables).

For peer review only

Reasons for non-adherence to medication and acceptability of an interactive voice response intervention in patients with hypertension and type 2 diabetes in primary care. A qualitative study

Katerina Kassavou and Stephen Sutton

Corresponding author:

Katerina Kassavou, BSc in Psychology, MSc in Clinical Health Psychology, PhD in Health Psychology. Research Associate, Behavioural Science Group, University of Cambridge, School of Clinical Medicine, Institute of Public Health and Primary Care, The Primary Care Unit, Forvie Site, Robinson Way, Cambridge, CB2 0SR. kk532@medschl.cam.ac.uk. +44(0)1223330456

Co-author:

Stephen Sutton, BSc in Combined Studies, Psychology main, MSc in Social Psychology, MSc in Computer Science, PhD in Social Psychology. Professor in Behavioural Science, University of Cambridge, Cambridge, UK.

Keywords or phrases: Medication adherence, interactive voice response, hypertension, type 2 diabetes.

Word count: 4,766 (excluding title page, abstract, references, figures and tables).

For peer review only

For peer review only - http://bmjopen.bmj.com/site/about/guidelines.xhtml

Acceptability of the Interactive Voice Response to support reasons for non-adherence to cardiovascular risk reduction medication

ABSTRACT

Objectives: The aim of this study was to explore the factors that impact on medication non-adherence and test the acceptability of an Interactive Voice Response (IVR) intervention to support patients with hypertension and/or type 2 diabetes, as an adjunct to primary care.

Design, method, participants and setting: The study included face-to-face interviews with 19 patients with hypertension and/or type 2 diabetes recruited from primary care databases and presumed to be non-adherent, 13 of these patients pre-tested elements of the IVR intervention few months later using a think aloud protocol. We also interviewed five practice nurses. A multi-perspective and longitudinal thematic analysis informed the findings.

Results: Beliefs about taking medications, the prescribed regimens and limited ability to cope with the underlying affective state within challenging contexts were mentioned as important reasons for medication non-adherence. Nurses reported time constraints to address each patient's different reasons for medication non-adherence and efficacy to support them between primary care consultations. Participants gave positive experiential feedback on the IVR intervention as a way to support them between their primary care consultations and provided recommendations for content and delivery features. Specifically, participants liked the voice delivering the messages and the voice recognition software. For intervention content, participants preferred messages that included tailored information about health consequences, action plans and simple reminders for performing the behaviour.

Conclusions: People with hypertension and/or type 2 diabetes and practice nurses suggested interventions tailored to each patient's reasons for non-adherence. Patients recommended IVR as an acceptable platform to support them take their medications between their primary care consultations. Future studies could usefully test the acceptability of tailored IVR interventions to support medication adherence in primary care.

For peer review only - http://bmjopen.bmj.com/site/about/guidelines.xhtml

Acceptability of the Interactive Voice Response to support reasons for non-adherence to cardiovascular risk reduction medication

Strength and limitations of this study

- Participants: patients presumed to be non-adherent to medications and nurses recruited by primary care practices from a range of different levels of deprivation.
- Analysis: multi-perspective analysis of patients and nurses' views on reasons for non-adherence and current action to address medication adherence.
- Design: development of a novel Interactive Voice Response intervention to support medication adherence and pre-tested intervention content and delivery mode using think aloud protocol.
- Applicability: consideration should be given when translating these results to different populations and settings.

For peer review only

Funding statement

"This work was supported by [name of funder] grant number [xxx]"

Acceptability of the Interactive Voice Response to support reasons for non-adherence to cardiovascular risk reduction medication

INTRODUCTION

Primary care consultations aim to advise and support patients in their decision to take their medications (1), however a substantial proportion of patients cease or do not take their medications as prescribed after leaving the consultation room (2, 3). This contributes to low treatment efficacy and increased NHS costs (4), particularly for people who use health care services regularly, such as people with type 2 diabetes and hypertension (2, 4, 5).

A theoretical framework categorises the causes of non-adherence into intentional and non-intentional reasons (6, 7). Intentional non-adherence (INA) refers to patients who consciously decide to miss or alter their medication regimens, whereas non intentional non-adherence (NINA) refers to patients who miss or alter their doses without conscious consideration of their behaviour. Currently we do not know how to effectively address patients' reasons for medication non-adherence (8). Both a NICE guideline (1) and a report commissioned by the Department of Health (4) recommend the development and test of novel interventions to address medication non-adherence.

Interventions using existing telephone systems are promising platforms to support patients between consultations. Our recent systematic review showed that telecommunication interventions can double the odds of increasing medication taking in 25,000 people with or at risk of cardiovascular conditions, when added or compared to usual care (9). Such interventions enable tailored behaviour change communication using automated voice (IVR) messages. In the UK adults use 216.8 billion of minutes in voice calls (142.8 billion via mobile phones and 74 billion minutes via fixed lines) every year (10, 11). Despite this major potential to reach a large number of patients and support medication adherence at a low cost (12), no such intervention has been developed and tested in the UK primary care setting.

In this study we aimed to identify the reasons for patients' non adherence to medications and assess the acceptability of an automated, telephone-based intervention to address these reasons and promote medication adherence. To achieve this, we explored non-adherent participants' views on a) the reasons they do not take their medications as prescribed; b) their actions to facilitate medication adherence; and c) their views and recommendations on an IVR intervention to support medication adherence, as an adjunct to practitioners' consultations.

For peer review only - http://bmjopen.bmj.com/site/about/guidelines.xhtml

METHODS

Recruitment

The study was approved by the NHS Ethics Committee and the NHS (*name*) Clinical Commissioning Group (CCG) before any participant was approached. Primary care practices within (*place*) with the highest recorded numbers of patients with hypertension and type 2 diabetes mellitus (T2DM) and at different levels of deprivation were eligible for this study. Practices were identified via the Public Health England website. Sixty one practices were invited and five of them participated in the study, of which four had list sizes smaller than the average and one larger than the average compared to other practices in the CCG. The practices varied in deprivation level. Three practices invited nurses and four invited patients in the study.

Nurses eligible for inclusion were those who were involved in advising, encouraging or supporting patients to adhere to their treatments. Eligible nurses were approached face-to-face by the practice manager, given the study information pack and invited into the study. Five practice nurses from three practices consented and took part in the interview.

Patients eligible for inclusion were those (a) aged 40 and over (b) with a primary diagnosis of hypertension and/or T2DM, (c) who were not adherent to their medication regimens, as indicated by clinical measures closest to the last refilled medication prescription or those with gaps in ordering or refilling repeat prescriptions during the last three months, and (d) had visited the practice at least once during the last six months.

The practice manager randomly selected eligible patients from the practice database. A practice GP screened the list of eligible patients for the main exclusion criteria (e.g. recent acute health event; diagnosis of dementia; aphasia or other cognitive difficulties; severely impaired hearing or speaking). Practice staff posted study invitation packs to those selected for inclusion. In total 150 invitations were sent to eligible patients and 19 expressed an interest to participate in the study. We decided to cease recruitment when saturation of the data had been achieved (13). We recruited fewer nurses than stated in the protocol, as the collected data had reached saturation after implementing constant comparisons between themes.

Interviews

For peer review only - http://bmjopen.bmj.com/site/about/guidelines.xhtml

At baseline, in-depth interviews were conducted using a semi-structured guide to identify the barriers to medication adherence in primary care, informed by previous research (14, 15). We asked nurses their views on: patients' experiences with medication non-adherence, their current practices to address non-adherence, and their recommendations on the development of a telecommunication intervention. We asked patients about their views on: their condition, medication-taking, barriers and facilitators in taking their medications, and whether and how a telecommunication intervention could support them take their medications as prescribed.

Based on the baseline interviews we developed an interactive voice response (IVR) system. The IVR messages were developed by combining the findings from the baseline interviews and a systematic literature review exploring the effective Behaviour Change Techniques (BCTs) to support medication adherence (9). At the follow up interviews, we used a think aloud protocol to gain participants' experiential feedback on elements of the IVR. Each IVR call lasted approximately 1-5 minutes. Participants were asked to trigger and complete one or two IVR calls and verbalise their thoughts during and after the calls. Participants were asked and prompted to think aloud their views on the delivery mode, the content of the messages, and their recommendations on how this intervention could be improved and support them best.

The first author constructed the interview guide, think aloud protocol, voice messages, and conducted interviews and analysis, with input from the second author. The first author had experience and training in qualitative research. The second author is also a leading expert in the topic of research. Patients were interviewed in their homes (n=11 baseline and n=12 at follow up), at the university (n=7 at baseline) or at their workplace (n=1 at baseline and n=1 at follow up). There was the intention for all the follow-up interviews to take place in participants' homes. All nurses were interviewed at their practices. All interviews were face-to-face with only the researcher and the interviewee present, apart from one baseline interview where the interviewee's partner was present to help with translation. Each baseline interview lasted on average 1 hour and each follow up interview about 30 minutes. All interviews were digitally recorded, transcribed by an independent service and double-checked with field notes for accuracy by the researcher.

Analysis

For peer review only - http://bmjopen.bmj.com/site/about/guidelines.xhtml

We analysed the data from the two cross-sectional sets of interviews (i.e. patients and nurses) using multi-perspective thematic analysis (16). All interviews were analysed separately and then data were integrated into broader descriptive categories. For baseline interviews, latent themes from patients and nurses' interviews were identified inductively and synthesised into one analysis, capturing both perspectives. Themes from the follow-up interviews were mapped onto those from baseline interviews and any new themes (e.g. on IVR acceptability) were treated separately (17).

RESULTS

Participants

In total 37 interviews were conducted: 19 patients were interviewed at baseline, 13 of whom were interviewed a few months later, and 5 nurses. Patients' mean age at baseline was 62.3 years, seven of them were female, thirteen reported as primary diagnosis hypertension, three reported T2DM and the rest reported diagnosis of co-morbidities of CVD (e.g. stroke) and T2DM (for practices, nurses and patients' characteristics see Table 1).

Table 1: Practice and participants' characteristics.

**Practice** (deprivation level within CCG, practice list size on average within CCG, area)	**Participants**					
	**Nurses' pseudonyms and gender**	**Patients**				
		**pseudonyms**	**age**	**gender**	**long-term condition**	**participated at follow up interview (reason if not)**
Average deprived, small, urban	A1 female	B1	80	male	DT2 & hypertension	Yes
		B2	70	male	hypertension (&stroke)	Yes
		B3	67	male	hypertension	yes
		B4	70	female	DT2	Not interested
		B5	66	male	hypertension	Not interested
		B6	71	female	hypertension	Yes
Least deprived, small, rural	A3 female	B7	57	female	hypertension	Moved area
	A4 female	B8	61	female	hypertension	Yes
Less deprived,		B9	51	female	hypertension	Yes

For peer review only - http://bmjopen.bmj.com/site/about/guidelines.xhtml

For peer review only

large, rural		B10	64	female	hypertension	Yes
		B11	70	male	hypertension & DT2	Yes
		B12	64	male	hypertension	Yes
		B13	65	male	hypertension	Yes
		B14	44	male	hypertension	No time
		B15	52	male	hypertension	Yes
		B16	61	male	hypertension and DT2	Yes
Most deprived, small, rural	A2 female	B17	45	male	hypertension	Excluded
		B18	50	female	DT2	Yes
Least deprived, small, rural	A5 female	B19	77	male	DT2	No time
		Mean age in years	62.3			

Reasons for medication non-adherence

Participants reported that people do not initiate taking their medications as prescribed when they do not understand the need to take medications to support them manage their long-term conditions.

Quote 1

Some participants reported that they intentionally changed or stopped the course of their medications when they had no health benefits from taking them. The underlying affective state associated with taking medications was important to mobilize continued medication adherence. Participants who reported not feeling any different by taking medications, seemed to report low motivation to adhere.

Quote 2

Alternatively, participants who had unpleasant side effects from taking medications reported stopping or skipping their tablets.

Quote 3

The regimens people were prescribed was also mentioned as an important barrier for medication non-adherence. People taking lots of pills reported limited ability to cope with multiple regimens, and those taking few pills reported lack of motivation to continue taking their prescribed tablets.

For peer review only - http://bmjopen.bmj.com/site/about/guidelines.xhtml

Quote 4

Some participants reported beliefs that behaviours can have an accumulative or counteractive effect on each other. In these cases, participants tended to alter their dosages according to the other behaviours enacted at times and/or places related to their mediation-taking behaviour. For example, people reported that they intentionally alter or miss their medications when socialising, especially when their social environment did not support them taking their medications.

Quote 5

Some participants also reported difficulties in taking their medications when they change their everyday routine and/or they are busy. For example, one participant reported forgetting taking his medications when going away for the weekend, whereas another reported forgetting when he has multiple activities to do in a day. Additionally, nurses reported participants' memory issues as a reason for forgetfulness.

Quote 6

Current actions to promote medication adherence and recommendations for an intervention

Participants reported that one way to remember to take their medications as prescribed was to add reminders to their environment. These cues were already associated with activities of their daily routine, and were enacted at close time and space proximity with taking medication.

Quote 7

Patients perceived current practices in primary care as satisfactory in terms of providing them with advice on how to take their medications. Some nurses also reported that they encourage patients to add cues to their environment to help them cope with forgetfulness. Another nurse also reported that she advises patients about how to cope with their underlying affective state during the course of their medications.

Quote 8

For peer review only - http://bmjopen.bmj.com/site/about/guidelines.xhtml

Acceptability of the Interactive Voice Response to support reasons for non-adherence to cardiovascular risk reduction medication

Both nurses and patients expressed favourable views on a telecommunication intervention and suggested that such an intervention could be more effective if it was tailored to each patient's reasons for not taking medications as prescribed.

Quote 9

However, both patients and nurses reported challenges with the time available within a consultation to address each patient's needs regarding their medications. Moreover, participants reported challenges with their abilities to support themselves (in the case of patients) or provide support to patients (in the case of nurses) to adhere to their prescriptions daily and/or during conflicting conditions.

Quote 10

Acceptability of the IVR delivery mode

IVR seemed to be an acceptable platform to deliver messages for medication adherence for the majority of the participants. Specifically, participants liked the voice delivering the call, the navigation options and the voice recognition software. Even one person, who was reluctant about the automatic element of the call, reported that receiving an automated call would have been better than forgetting medications.

Quote 11

Participants seemed to also like the interactive element of the IVR delivery mode, which they used to report missing some of their dosages and/or to ask for advice on how to cope with non-adherence.

Quote 12

Acceptability of the intervention content

Most participants expressed a favourable view on voice messages including information about health consequences, especially since hypertension and/or T2DM can be asymptomatic. Specifically, participants highlighted the importance of messages that reminded them of the reasons they need to take their medications. Participants reported that receiving the messages

For peer review only - http://bmjopen.bmj.com/site/about/guidelines.xhtml

at occasions they usually find challenging taking their medications could support them taking their tablets as prescribed.

Quote 13

Additionally, most participants liked simple messages that prompted them to take their medications at a due time and did not require conscious thinking on their part.

Quote 14

However, messages that focused on information about the duration of their health condition rather than ways to support them take their medications and messages asking them to add effort in self-monitoring their medication taking behaviour (e.g. keeping a diary every day) seemed not to be acceptable.

Quote 15

Personalised voice messages (i.e. that included patients' names) seemed to be satisfactory to the majority of participants.

Quote 16

DISCUSSION

Principal findings

Beliefs about the need to take medications, the number of prescribed doses and the limited ability to cope with the underlying affective state within challenging contexts were mentioned as important reasons for medication non-adherence. Nurses reported time constraints to address each patient's different reasons for medication non-adherence and efficacy to support them between and sometimes during the primary care consultations. Both patients and nurses had favourable views on an automated intervention and recommended messages to be tailored to each patient's needs for taking medications. Patients expressed positive experiential feedback on the IVR and provided recommendations for content and delivery features. Specifically, participants liked the voice delivering the messages and the voice recognition software. For intervention content, patients expressed a preference for messages with tailored information about health consequences and simple medication reminders. The

For peer review only - http://bmjopen.bmj.com/site/about/guidelines.xhtml

interactive element of the interventions seemed to be most useful for people to ask support when they had missed their tablets. Including elements of personalisation would increase uptake and initial engagement with the intervention.

Strengths and limitations

In this study we invited patients whose practice records suggested that they were not refilling their prescriptions and/not managing their high blood pressure. Insights obtained from these patients can usefully inform the content and delivery of a medication adherence intervention within primary care. However, consideration should be given when translating these results to different populations (e.g. patients who do not attend practices or those not participating in research) and other settings (e.g. community).

Comparison with existing literature

Previous studies have investigated the reasons for medication non-adherence (18), as well as patients or practitioners' views of an intervention to address non-adherence (19, 20). In accordance with previous research, participants' attitudes about the need to take medications, especially when they take multiple medications (21) seem to be important for medication non-adherence. Similarly, previous intervention development and feasibility studies suggested that telecommunication interventions should remind people about their medications prescription plan, and provide patients with the options of interactive communication and tailoring of the intervention content (22, 23). However, none of these studies had investigated the underlying determinants that account for medication non-adherence, how current practices address these, or considered multiple views and recommendations on intervention delivery mode and content to support maintained adherence to medications. Most importantly, none of the previous studies have investigated the acceptability of an IVR intervention to address medication non-adherence in the UK primary care setting, which is an important part of developing novel interventions (24).

Implications for research and practice

Within the time constraints of a primary care consultation, practitioners could briefly address patients' main reasons for non-adherence (INA and/or NINA) and then signpost patients to a telecommunication intervention designed to support adherence between practice visits. IVR seems to be an acceptable mode to provide advice and support using patients' telephones.

For peer review only - http://bmjopen.bmj.com/site/about/guidelines.xhtml

Moreover, patients reported medication-taking reminders and tailored information about health consequences as acceptable content to promote medication adherence.

Unanswered questions and future research

Future studies aiming to develop such interventions in primary care could usefully tailor the intervention to each patient's needs by mapping the voice messages to their reasons for medication non-adherence. Moreover, future studies could integrate participants' views on barriers and facilitators to uptake, retention and engagement with such interventions. Participants' views and recommendations on the delivery mode, such as the clarity, tone, volume, intensity, speed rate, rhythm, and vocal expression of the messages, the navigation options and the frequency and duration of the calls should be explored. Moreover, future studies could explore participants' views, satisfaction, mastery and actions upon the content of the voice messages, and obtain recommendations for improvement.

Authors' contributions and competing interests

SS and KK designed the study. KK conducted all tasks involved in completing this study, with substantial input from SS. Authors have no competing interests.

Data sharing statement

Documents describing the process and the data of analysis are available by request.

REFERENCES

1. National Institute of Health and Care Excellence. Medicine adherence. Involving patients in decisions about prescribed medicines and supporting adherence 2009.

2. World Health Organisation. Adherence to long-term therapies. Evidence for action. 2003.

3. Chowdhury R, Khan H, Heydon E, et al. Adherence to cardiovascular therapy: a meta-analysis of prevalence and clinical consequences. *Eur Heart J* 2013;34(38):2940-8.

4. Department of Health. Improving the use of medicines for better outcomes and reduced waste. An action plan. 2012.

For peer review only - http://bmjopen.bmj.com/site/about/guidelines.xhtml

5. Bailey JE, Wan JY, Tang J, et al. Antihypertensive medication adherence, ambulatory visits, and risk of stroke and death. *J Gen Intern Med* 2010;25(6):495-503.

6. Barber N. Should we consider non-compliance a medical error? *Qual Saf Health Care* 2002;11(1):81-4.

7. Marshall IJ, Wolfe CD, McKevitt C. Lay perspectives on hypertension and drug adherence: systematic review of qualitative research. *BMJ* 2012;345:e3953.

8. Nieuwlaat R, Wilczynski N, Navarro T, et al. Interventions for enhancing medication adherence. *Cochrane Database Syst Rev* 2014;11:CD000011.

9. XX, XX. What are the effective Behaviour Change Techniques in brief automated telecommunication interventions to promote adherence to cardiovascular risk reduction medications? A systematic review and meta-regression (*under review*).

10. Statistica. Number of mobile voice call minutes in the United Kingdom (UK) from 2007 to 2015 (in billion minutes). 2016.

11. Statistica. Number of fixed voice call minutes in the United Kingdom (UK) from 2007 to 2015 (in billion minutes). 2016.

12. Smith DH, O'Keeffe-Rosetti M, Owen-Smith AA, et al. Improving Adherence to Cardiovascular Therapies: An Economic Evaluation of a Randomized Pragmatic Trial. *Value Health* 2016;19(2):176-84.

13. Chamberlain K, Camin P, Yardley L. Qualitative analysis of experience: grounded theory and case studies. In: Marks D, Yardley L, editors. Research Methods for Clinical and Health Psychology. London SAGE publications; 2004.

14. Farmer A, Hardeman W, Hughes D, et al. An explanatory randomised controlled trial of a nurse-led, consultation-based intervention to support patients with adherence to taking glucose lowering medication for type 2 diabetes. *BMC Fam Pract* 2012;13:30.

15. Sutton S. Stage theories. In: Norman MCP, editor. Predicting and changing health behaviour: Research and practice with social cognition models 9. Buckingham: Open University Press; 2015.

16. Kendall M, Murray SA, Carduff E, Worth A, Harris F, Lloyd A, et al. Use of multiperspective qualitative interviews to understand patients' and carers' beliefs, experiences, and needs. *BMJ* 2009;339:b4122.

17. Kassavou A, Turner A, French DP. The role of walkers' needs and expectations in supporting maintenance of attendance at walking groups: a longitudinal multi-perspective study of walkers and walk group leaders. *PLoS One* 2015;10(3):e0118754.

18. De Simoni A, Mant J, Sutton S. Adherence to medication in stroke survivors dependent on caregivers. *British J Gen Pract* 2015;65(640):e789-91.

19. Lecouturier J, Cunningham B, Campbell D, et al. Medication compliance aids: a qualitative study of users' views. *British J Gen Pract* 2011;61(583):93-100.

For peer review only - http://bmjopen.bmj.com/site/about/guidelines.xhtml

20. Nolan T, Dack C, Pal K, et al. Patient reactions to a web-based cardiovascular risk calculator in type 2 diabetes: a qualitative study in primary care. *British J Gen Pract* 2015;65(632):e152-60.

21. Jamison J, Graffy J, Mullis R, et al. Stroke survisors', caregivers' and GPs' attitudes towards a polypill for the secondary prevention of stroke: a qualitative interview study. *BMJ Open* 2016; **6**:e010458 doi:10.1136/bmjopen-2015-010458

22. Rana Y, Haberer J, Huang H, et al. Short message service (SMS)-based intervention to improve treatment adherence among HIV-positive youth in Uganda: focus group findings. *PLoS One* 2015;10(4):e0125187.

23. Osborn CY, Mulvaney SA. Development and feasibility of a text messaging and interactive voice response intervention for low-income, diverse adults with type 2 diabetes mellitus. *J Diabetes Sci Technol 2013*;7(3):612-22.

24. Craig P, Dieppe P, Macintyre S, et al. Developing and evaluating complex interventions: the new Medical Research Council guidance. *BMJ* 2008;50(5):587-92.

For peer review only

For peer review only

For peer review only - http://bmjopen.bmj.com/site/about/guidelines.xhtml

Reasons for non-adherence to medication and acceptability of an interactive voice recognition intervention in patients with hypertension and type 2 diabetes within the primary care. A qualitative study

No	Quote
1	"*I think probably they don't think they need them to be honest with you. 'I don't need them what are you giving me these for' yeah 'I'm well look at me, I've been like this all me life*" patient B2<br>"*the trouble with diabetes is people don't realise that they've got it and they go on for a couple of years before it's discovered and they probably have gone all this time and not felt ill at all and then they've been given a tablet so they haven't really had a time when they felt ill*" patient B4<br>"*I suppose, deep down are not really happy to be taking medication and they think if they ignore the problem then, or they keep the tablets on the side and they think 'oh I'll take it if I really need it*' nurse A5
2	"*He say that he take one tablet one day, two tablets, second tablet second day and it help but after for example after second tablet on the fourth day it don't help any more ….[he became] accustomed with this tablet with the medications [and don't want to take more]. He went more times to the GP and they gave the same. The tablets did nothing*" patient B17<br>"*I don't really ever consider that I've got high blood pressure. I just don't think about it… with blood pressure it's just how I feel I don't know what it feels like. It is, like you say there's nothing really to tell me day to day* " patient B14<br>"*I often think all these six going down my throat all in one go and they're all releasing their selves but I don't feel any different no...You don't realise what these tiny little things have got in them do you?*" patient B4<br>"*If they're feeling well they'll drop it out or, drop down … they think they don't need it .. They just don't want to take something every day*" nurse A4
3	"*the side effects there were which made me feel ill, there's no point in taking pills that make you feel ill is there?*" patient B2<br>"*I felt as though I was walking through treacle, I wasn't right at all so no we don't take that one anymore*" patient B10<br>"*I'll come across people who won't take something the doctor has prescribed because they're getting side effects that they don't like. For example Metformin which gives them tummy ache and the doctor might have prescribed say two or three doses a day and they'll only take one...generally speaking Metformin in the first couple of weeks can cause a lot of indigestion, nausea, tummy aches that kind of thing and often patients don't like it, so they stop taking it*" nurse A5
4	"*If I'm routinely asked to take a pill without really knowing why I'm taking it, it's not a very good incentive to take that pill…. they may walk out of this surgery thinking 'do I really need to take this [medication]?', lose the prescription or not quite make it to the chemist*" patient B13<br>"*Sometimes I think it's that they, they've got so much medication, they say 'I've got so much*

For peer review only

	medication I forget'" nurse A3
5	"*The only time I don't take it, if I go to a birthday party or wedding or whatever where you abuse yourself with a bit too much alcohol, I think I'd rather not take the tablets because I don't want it interfering with the alcohol in me, there's enough poison in there as it is"* patient B11<br>*"Yeah, I won't take it if I'm out, if we go out for dinner I won't take my tablets because I don't want other people to see me taking them*" patient B18
6	"*I just take them when I get up and if I get up in a rush then that is when I can forget. I will take the cardinipine later in the day but if I've remembered I've forgotten after about lunchtime*" patient B8<br>"*Oh well I got down to me daughter's in Kent once, when was that? Not last Christmas, the Christmas before and I'd forgotten, I'd taken most of me pills but I'd forgotten half of them so we had to go round to the local doctors and get a prescription, luckily I'd got a copy of my prescription what I take*" patient B1<br>"*memory problems, so we would see maybe forgetfulness, living on their own nobody to prompt them*" nurse A1<br>"*I came across a patient the other day who admitted that he took it when he remembered and that was possibly two, three times a week*" nurse A5
7	"*[In the morning] I have a coffee I put the mug of milk [for the coffee] in the microwave…before I go to bed I put the pills in the microwave…to make me remember and get into the habit of it, you don't have your coffee until you've had your medication otherwise you don't have a coffee. You must associate that and put them together, I do and it works*" patient B3<br>"*I suppose it's quite a little routine isn't it? I actually put a handkerchief by the bed so that I wake up either when the alarm goes or usually I wake up half an hour or so before the alarm and then doze and I find it and take it with a shot of water, I have a bottle of water by the bed*" patient B5<br>"*I think it's about patterns in my life so every morning I have a tray, that's where my phone, my keys are, everything I need for the next day I put on that tray ... so every morning I'd put that [tablet] on my little tray, so really just finding somewhere every day where I've got the things that I need for the next day has been a help to me. Because when I did not have that pattern I'd go to work and 'oh I need my pill now, it's at home'*" patient B14
8	"*Just a lot of encouragement, there was a lot of contact and checking 'have you done this' and 'did you do that' or 'well done you', trying to give them a bit of a boost. I think that's about the best you can do*" patient B10<br>"*I said to her 'would you go out the house without cleaning your teeth', 'no', 'so put it by your toothbrush*" nurse A1<br>"*The other thing I tell them is that how you think you feel is often not a good indication of*

For peer review only - http://bmjopen.bmj.com/site/about/guidelines.xhtml

	actually what state you are in because you just get used to feeling in a certain way" nurse A3
9	"*It's like there's not a pill that will sort everyone out. It's a very complicated thing you really need to fit the answer to the person so you need to find out from them… in order to get someone to take their medication you've got to understand how they fit their world together…so if someone doesn't establish routines you've got to find out how they're keeping their life together*" patient B5<br>"*One size doesn't fit all really does it? Everybody's different and what works for one person doesn't always work for another… ask them what they would prefer I think that could be good*" patient B9<br>"*I think it would need to be different for different people I don't think you could do that across the board*". nurse A5
10	"*I think if you've been advised to take it and you don't what can they do? I don't see what help they can do*" patient B6<br>"*I had a new patient's appointment on Tuesday afternoon and she's got a set list of things that she's got to do, she's got to go through what medication you're on, she's got to test your urine, she's got to take your blood pressure and ask these questions and she's got 10 minutes to do it*" patient B9<br>"*Yeah on the prescriptions it will say "take one tablet daily or every day"… people don't want to sit and read it*" nurse A1<br>"*They don't actually understand how the stuff works in the first place so but, but I know that I've had patients back who are saying 'I didn't know that' and I know that I told them it two years ago because it's documented in their notes so they don't always listen either that's the other thing… But we really haven't got time to be going into everybody's medications and why and how they take them.. I just think you can't go round people's houses and ram their pills down their throat twice a day can you*" nurse A4<br>"*Sometimes patients say things to you and then you find out when you actually check their medications or prescriptions that they're not doing what you asked them to do really .. and I suppose there's always this feeling of 'I'll do something about it' very motivated, leave the surgery, 'I'll sort it out, not going to have medicines I'm going to sort this problem out' but actually when it comes to it, it's very difficult to change some lifestyle*" nurse A5
11	"*Now that you've told me it's not a person I'm talking to I can say what I like can't I?..A lot of people when they pick up that phone are going to think they are talking to a real person. They're not. It's a nice, it's well pitched, the lady is a pleasant voiced lady, it doesn't sound like a cold call or anything…[voice recognition] it's better than pressing buttons, I hate pressing buttons it's awful*" patient B13<br>"*I don't know how to explain it but to be actual talk to a person than a machine is entirely*

For peer review only - http://bmjopen.bmj.com/site/about/guidelines.xhtml

	different … that might put certain people off but on the other hand it might think 'thank goodness that came through I'll take my medication'" patient B6<br>"*So many firms if you press this and you press that, press one, press two, press three. I: Would you prefer that or would you prefer to speak to a (?) R: Yeah this is better, that's much better*" patient B8
12	"*If you've got something wrong with you then you probably wait a few months to go to the doctors…or they'll think 'oh I can get over this' and so on but really in the background in their mind they are slightly worried and they might want a bit more information. I: So do you think they would do that through that system? R: I think they would as a starting point because it's not going, it's not talking on a one to one with somebody, you're actually just accessing an accurate database which is reliable* " patient B16<br>"*I did forget to take my tablets this morning, can I still take them now or should I just wait for the next day?"* patient B18 inbound call<br>"*if I ring up now and say 'oh my prescription's run out can I order a new prescription?"* patient B12
13	"*R: people get it into their heads that they feel alright and therefore they don't need to take it. I thought it was good in the message that you said that they need to take it regularly in order to maintain, to keep their blood pressure down.*<br>*I: do you think this information is not known to people?*<br>*R: maybe they choose to ignore it. They probably do but high blood pressure is one of those things that unless you actually feel it you're not aware that it's a problem*" patient B9
14	"*Hello Mrs, have you taken your drugs today" just to remind them*" patient B2<br>"*I'm thinking if they ring up, it's like some of my medication like the warfarin I have to take it at a certain time if I don't take it then I've basically got to wait 24 hours and take the next lot. If it says 'have you taken your medication' you say 'no' then that comes across and say 'oh best I'd take it'*" patient B12<br>"*I got a call at midday saying 'have you taken your medications today?' …it would cause me to think before I answered, [laughs]"* patient B16
15	"*It's saying to me, you've been taking this medication for seven years. I don't need them to tell me why, like I know really I just need reminding to take it. The other thing is it says about writing down in your diary but if I was getting the calls I would be like 'what are you talking about?' do you know what I mean? I wouldn't want to be told to write it down. I think if I was that organised that I would write down in a diary I wouldn't need somebody to ring me. I literally would just want it to say 'take your medicine'*" patient B18
16	"*I think they will because it says your name so you're waiting for it* " patient B18<br>"*It feels like somebody who's closer to you, somebody's who's on, familiar with you so in that*

For peer review only - http://bmjopen.bmj.com/site/about/guidelines.xhtml

	respect it's better" patient B9

For peer review only

COREQ (COnsolidated criteria for REporting Qualitative research) Checklist

A checklist of items that should be included in reports of qualitative research. You must report the page number in your manuscript where you consider each of the items listed in this checklist. If you have not included this information, either revise your manuscript accordingly before submitting or note N/A.

Topic	Item No.	Guide Questions/Description	Reported on Page No.
**Domain 1: Research team and reflexivity**			
Personal characteristics			
Interviewer/facilitator	1	Which author/s conducted the interview or focus group?	6
Credentials	2	What were the researcher's credentials? E.g. PhD, MD	1
Occupation	3	What was their occupation at the time of the study?	1
Gender	4	Was the researcher male or female?	1
Experience and training	5	What experience or training did the researcher have?	6
Relationship with participants			
Relationship established	6	Was a relationship established prior to study commencement?	5 - see patients
Participant knowledge of the interviewer	7	What did the participants know about the researcher? e.g. personal goals, reasons for doing the research	5
Interviewer characteristics	8	What characteristics were reported about the inter viewer/facilitator? e.g. Bias, assumptions, reasons and interests in the research topic	1
**Domain 2: Study design**			
Theoretical framework			
Methodological orientation and Theory	9	What methodological orientation was stated to underpin the study? e.g. grounded theory, discourse analysis, ethnography, phenomenology, content analysis	6,7
Participant selection			
Sampling	10	How were participants selected? e.g. purposive, convenience, consecutive, snowball	5
Method of approach	11	How were participants approached? e.g. face-to-face, telephone, mail, email	5
Sample size	12	How many participants were in the study?	7
Non-participation	13	How many people refused to participate or dropped out? Reasons?	5, 7, 8
Setting			
Setting of data collection	14	Where was the data collected? e.g. home, clinic, workplace	6
Presence of non-participants	15	Was anyone else present besides the participants and researchers?	6
Description of sample	16	What are the important characteristics of the sample? e.g. demographic data, date	7,8
Data collection			
Interview guide	17	Were questions, prompts, guides provided by the authors? Was it pilot tested?	6
Repeat interviews	18	Were repeat inter views carried out? If yes, how many?	6, 7
Audio/visual recording	19	Did the research use audio or visual recording to collect the data?	6
Field notes	20	Were field notes made during and/or after the inter view or focus group?	6
Duration	21	What was the duration of the inter views or focus group?	6
Data saturation	22	Was data saturation discussed?	5
Transcripts returned	23	Were transcripts returned to participants for comment and/or	no

For peer review only

For peer review only - http://bmjopen.bmj.com/site/about/guidelines.xhtml

Topic	Item No.	Guide Questions/Description	Reported on Page No.
		correction?	
**Domain 3: analysis and findings**			
Data analysis			
Number of data coders	24	How many data coders coded the data?	6
Description of the coding tree	25	Did authors provide a description of the coding tree?	no
Derivation of themes	26	Were themes identified in advance or derived from the data?	6-7
Software	27	What software, if applicable, was used to manage the data?	n/a
Participant checking	28	Did participants provide feedback on the findings?	6,7
Reporting			
Quotations presented	29	Were participant quotations presented to illustrate the themes/findings? Was each quotation identified? e.g. participant number	8-11
Data and findings consistent	30	Was there consistency between the data presented and the findings?	8-11
Clarity of major themes	31	Were major themes clearly presented in the findings?	8-11
Clarity of minor themes	32	Is there a description of diverse cases or discussion of minor themes?	10

Developed from: Tong A, Sainsbury P, Craig J. Consolidated criteria for reporting qualitative research (COREQ): a 32-item checklist for interviews and focus groups. *International Journal for Quality in Health Care*. 2007. Volume 19, Number 6: pp. 349 – 357

Once you have completed this checklist, please save a copy and upload it as part of your submission. DO NOT include this checklist as part of the main manuscript document. It must be uploaded as a separate file.

For peer review only

BMJ Open

Reasons for non-adherence to medication and acceptability of an interactive voice response intervention in patients with hypertension and type 2 diabetes in primary care. A qualitative study.

Journal:	*BMJ Open*
Manuscript ID	bmjopen-2016-015597.R1
Article Type:	Research
Date Submitted by the Author:	27-Apr-2017
Complete List of Authors:	Kassavou, Katerina; University of Cambridge, Public Health and Primary Care ; University of Cambridge,<br>Sutton, Stephen; University of Cambridge,
**Primary Subject Heading**:	Cardiovascular medicine
Secondary Subject Heading:	Communication, Diabetes and endocrinology, Health policy, Health services research, Medical education and training
Keywords:	Medication adherence, interactive voice response, Hypertension < CARDIOLOGY, Telemedicine < BIOTECHNOLOGY & BIOINFORMATICS, DIABETES & ENDOCRINOLOGY

SCHOLARONE™
Manuscripts

For peer review only

Reasons for non-adherence to medication and acceptability of an interactive voice response intervention in patients with hypertension and type 2 diabetes in primary care. A qualitative study

Aikaterini Kassavou and Stephen Sutton

Corresponding author:

Aikaterini Kassavou, BSc in Psychology, MSc in Clinical Health Psychology, PhD in Health Psychology. Research Associate, Behavioural Science Group, University of Cambridge, School of Clinical Medicine, Institute of Public Health and Primary Care, The Primary Care Unit, Forvie Site, Robinson Way, Cambridge, CB2 0SR. kk532@medschl.cam.ac.uk. +44(0)1223330456

Co-author:

Stephen Sutton, BSc in Combined Studies, Psychology main, MSc in Social Psychology, MSc in Computer Science, PhD in Social Psychology. Professor in Behavioural Science, University of Cambridge, Cambridge, UK.

Keywords or phrases: Medication adherence, interactive voice response, hypertension, type 2 diabetes.

Word count: 4,969 (excluding title page, abstract, references, figures and tables).

For peer review only

For peer review only - http://bmjopen.bmj.com/site/about/guidelines.xhtml

Acceptability of the Interactive Voice Response to support reasons for non-adherence to cardio-metabolic medications.

ABSTRACT

Objectives: This study explored the reasons for patients' medication non adherence, and tested the acceptability of an Interactive Voice Response (IVR) intervention to address non adherence to prescribed medicines, as an adjunct to primary care.

Design, method, participants and setting: The study included face-to-face interviews with 19 patients with Hypertension and/or type 2 Diabetes Mellitus randomly selected from primary care databases and presumed to be non-adherent. Thirteen of these patients pre-tested elements of the IVR intervention few months later using a think aloud protocol. Five practice nurses were interviewed. Data were analysed using a multi-perspective and longitudinal thematic analysis.

Results: Negative beliefs about taking medications, the complexity of prescribed medication regimens and the limited ability to cope with the underlying affective state within challenging contexts were mentioned as important reasons for non-adherence. Nurses reported time constraints to address each patient's different reasons for medication non adherence and limited efficacy to support patients between primary care consultations. Patients gave positive experiential feedback about the IVR messages as a way to support them take their medicines and provided recommendations for intervention content and delivery mode. Specifically, they liked the voice delivering the messages and the voice recognition software. For intervention content, they preferred messages that included tailored information about health consequences, action plans and simple reminders for performing the behaviour.

Conclusions: Patients with Hypertension and/or type 2 Diabetes and practice nurses suggested messages tailored to each patient's reasons for non-adherence. Participants recommended IVR as an acceptable platform to support adherence to medications between primary care consultations. Future studies could usefully test the acceptability of tailored IVR interventions to support medication adherence in primary care.

For peer review only - http://bmjopen.bmj.com/site/about/guidelines.xhtml

Acceptability of the Interactive Voice Response to support reasons for non-adherence to cardio-metabolic medications.

Strength and limitations of this study

- Participants: patients presumed to be non-adherent to medications and nurses recruited by primary care practices from different levels of deprivation.
- Analysis: multi-perspective analysis of patients and nurses' views on reasons for medication non adherence and current action to address reasons for non-adherence.
- Design: development of a novel Interactive Voice Response intervention to support medication adherence and pre-tested intervention content and delivery mode using think aloud protocol.
- Applicability: consideration should be given when translating these results to different populations and settings.

For peer review only

Funding statement. This paper presents independent research funded by the NIHR xxx (Grant Reference Number: xx). The views expressed are those of the author(s) and not necessarily those of the NHS, the NIHR or the Department of Health.

For peer review only - http://bmjopen.bmj.com/site/about/guidelines.xhtml

Acceptability of the Interactive Voice Response to support reasons for non-adherence to cardio-metabolic medications.

INTRODUCTION

Primary care consultations aim to advise and support patients in their decision to take their medications (1), however a substantial proportion of patients cease or do not take their medications as prescribed after leaving the consultation room (2, 3). This contributes to low treatment efficacy and increased NHS costs (4), particularly for people who use health care services regularly, such as patients with either Type 2 Diabetes Mellitus (T2DM) or Hypertension, or those with both health conditions (2, 4, 5).

The reasons for medication non adherence can be intentional or non-intentional (6, 7). Intentional non-adherence (INA) refers to patients who consciously decide to miss or alter their medication regimens, whereas non intentional non-adherence (NINA) refers to patients who miss or alter their doses without conscious consideration of their behaviour. Currently we do not know how to effectively address patients' reasons for medication non adherence (8), and practitioners' time is expensive and limited. Both NICE guidelines (1), and a report commissioned by the Department of Health (4), recommend the development and test of novel interventions to address non-adherence.

Interventions using existing telephone systems are promising platforms to support patients between consultations (9, 10). Our recent systematic review found that automated telephone-based interventions can double the odds to promote medication adherence, when added or compared to usual care (11). Such interventions enable behaviour change using automated voice (IVR) messages. In the UK adults use 216.8 billion of minutes in voice calls (142.8 billion via mobile phones and 74 billion minutes via fixed lines) every year (12, 13). Despite the major potential to reach a large number of patients and facilitate the delivery of low cost medication adherence services (14), no such intervention has been developed and tested in the UK primary care setting.

To address this gap, this study aimed to develop and assess the acceptability of an automated, telephone-based intervention to support medication adherence in patients with Hypertension and/or T2DM. To achieve this, the present study identified the reasons for patients' non adherence to medicines, the current actions to facilitate adherence, and obtained views and recommendations about an IVR intervention to influence the determinants of medication adherence, as an adjunct to primary care consultations.

For peer review only - http://bmjopen.bmj.com/site/about/guidelines.xhtml

For peer review only

Acceptability of the Interactive Voice Response to support reasons for non-adherence to cardio-metabolic medications.

METHODS

Recruitment

The study was approved by the NHS Ethics Committee (REC reference: 14/LO/1872) and the NHS Cambridgeshire and Peterborough Clinical Commissioning Group (CCG) before any participant was approached. Primary care practices within the CCG with the highest recorded numbers of patients with Hypertension and T2DM and at different levels of deprivation were eligible for this study. Practices were identified via the Public Health England website. Sixty one practices were invited and five of them participated in the study, of which four had list sizes smaller than the average and one larger than the average compared to other practices within the CCG. The practices varied in deprivation level. Three practices invited nurses and four invited patients to take part in the study.

Nurses eligible for inclusion were those who were involved in advising, encouraging or supporting patients to adhere to their treatments. Eligible nurses were approached face-to-face by the practice manager, given the study information pack and invited into the study. Five nurses from three practices provided written informed consent and took part in the interview.

Patients eligible for inclusion were those: (a) aged 40 and over, (b) with a primary diagnosis of Hypertension and/or T2DM, (c) who were non-adherent to their medication regimens, as indicated by clinical measures closest to the last refilled medication prescription or those with gaps in ordering or refilling repeat prescriptions during the last three months, and (d) had visited the practice at least once during the last six months.

The practice manager randomly selected eligible patients from the practice database. A practice GP screened the list of eligible patients for the main exclusion criteria (e.g. recent acute health event; diagnosis of dementia; aphasia or other cognitive difficulties; severely impaired hearing or speaking). Practice staff posted study invitation packs to those selected for inclusion. In total 150 invitations were sent to eligible patients and 19 expressed an interest and provided written informed consent to participate in the study. We decided to cease recruitment when saturation of the data had been achieved (15). We recruited fewer nurses than stated in the protocol, as the collected data had reached saturation after implementing constant comparisons between themes.

For peer review only - http://bmjopen.bmj.com/site/about/guidelines.xhtml

Acceptability of the Interactive Voice Response to support reasons for non-adherence to cardio-metabolic medications.

Interviews

At baseline, in-depth interviews were conducted using a semi-structured guide to identify the reasons of medication non adherence. The interview guide was informed by a review of evidence and theory based research (e.g. 16, 17). We asked nurses their views on: patients' experiences with taking medicines and barriers to adherence, their current actions to address non-adherence, and their recommendations about the development of telephone-based intervention, as an adjunct to primary care. Interviews with nurses were conducted from January until April 2015. We asked patients about their views on: their condition, medication adherence, barriers and facilitators in taking their medicines, and whether and how an automated, telephone-based intervention could support them take their medications as prescribed. Baseline interviews with patients were conducted from July until September 2015.

Based on the baseline interviews we developed an interactive voice response (IVR) platform to deliver the intervention messages (9). The IVR messages were developed by combining the findings from the baseline interviews and a systematic literature review exploring the effective Behaviour Change Techniques (BCTs) to support medication adherence (11). At the follow up interviews, we used a think aloud protocol to gain participants' experiential feedback on elements of the IVR. Participants were asked to trigger and complete one or two IVR calls and verbalise their thoughts during and after the calls. Each IVR call lasted approximately 1-5 minutes. Participants were asked and prompted to think aloud their views on the delivery mode, the content of the messages, and their recommendations on how IVR messages could be improved and support them to adhere to their prescriptions best. Follow up interviews with patients were conducted from March until April 2016.

The first author constructed the interview guide, think aloud protocol, voice messages, and conducted interviews and analysis, with input from the second author. The first author had experience and training in qualitative research. The second author is also a leading expert in the topic of research. Patients were interviewed at their homes (n=11 baseline and n=12 at follow up), at the university (n=7 at baseline) or at their workplace (n=1 at baseline and n=1 at follow up). There was the intention for all the follow-up interviews to take place at participants' homes to facilitate the collection of contextual information (18). All nurses were

For peer review only - http://bmjopen.bmj.com/site/about/guidelines.xhtml

interviewed at their practices. All interviews were face-to-face with only the researcher and the interviewee present, apart from one baseline interview where the interviewee's partner was present to assist with translation. Each baseline interview lasted on average 1 hour and each follow up interview about 30 minutes. All interviews were digitally recorded and transcribed by an independent service. Transcripts were double-checked against audio recording and field notes for accuracy by the researcher.

Analysis

We analysed the data from the two cross-sectional sets of interviews (i.e. baseline interview with patients and nurses) using multi-perspective thematic analysis (19). All interviews were analysed separately. After that, data were integrated into broader descriptive categories. For baseline interviews, latent themes from patients and nurses' interviews were identified inductively and synthesised into one analysis, capturing both perspectives. Themes from the follow-up interviews were mapped onto those from baseline interviews and any new themes (e.g. on acceptability of the IVR delivery mode) were treated separately (20).

RESULTS

Participants

In total 37 interviews were conducted: 19 patients were interviewed at baseline, 13 of whom were interviewed few months later, and 5 nurses. Patients' mean age at baseline was 62.3 years, seven of them were female, thirteen reported as primary diagnosis Hypertension, three reported T2DM and the rest reported diagnosis of co-morbidities of CVD (e.g. stroke) and T2DM. More information about the practices, nurses and patients' characteristics can be found at Table 1.

For peer review only - http://bmjopen.bmj.com/site/about/guidelines.xhtml

For peer review only

Acceptability of the Interactive Voice Response to support reasons for non-adherence to cardio-metabolic medications.

Table 1: Practice and participants' characteristics.

**Practice** Deprivation level within CCG, area, practice list size on average within CCG, patient/doctor ratio, patient/staff ratio (excluding doctors).	**Participants**					
		**Patients**				
	**Nurses' pseudonyms and gender**	**pseudonyms**	**age**	**gender**	**long-term condition**	**participated at follow up interview (reason if not)**
Average deprived; urban; small; 1136.6/1; 516.6/1	A1 female	B1	80	male	T2DM & hypertension	Yes
		B2	70	male	hypertension (&stroke)	Yes
		B3	67	male	hypertension	yes
		B4	70	female	T2DM	Not interested
		B5	66	male	hypertension	Not interested
		B6	71	female	hypertension	Yes
Least deprived; rural; small; 1208/1; 302/1	A3 female	B7	57	female	hypertension	Moved area
	A4 female	B8	61	female	hypertension	Yes
Less deprived; rural; large; 1275.37/1; 351.8/1		B9	51	female	hypertension	Yes
		B10	64	female	hypertension	Yes
		B11	70	male	hypertension & T2DM	Yes
		B12	64	male	hypertension	Yes
		B13	65	male	hypertension	Yes
		B14	44	male	hypertension	No time
		B15	52	male	hypertension	Yes
		B16	61	male	hypertension and T2DM	Yes
Most deprived; urban; small; 702.6/1; 301/1	A2 female	B17	45	male	hypertension	Excluded
		B18	50	female	T2DM	Yes
Least deprived; rural; small; 1378/1; 393.8/1	A5 female	B19	77	male	T2DM	No time
		Participants mean age in years	62.3			

For peer review only - http://bmjopen.bmj.com/site/about/guidelines.xhtml

Acceptability of the Interactive Voice Response to support reasons for non-adherence to cardio-metabolic medications.

Average list size CCG N=933,265 of whom n=117,784 patients with hypertension and n=43,296 with diabetes.

Note: CCG: clinical commissioning group; A(x) letter code to anonymise nurses (number code to anonymise nurses); B(y) letter code to anonymise patients (number code to anonymise patients); T2DM: Type 2 Diabetes Mellitus.

Reasons for medication non-adherence

Participants reported that people do not initiate taking their medications as prescribed when they do not understand the need to take medicines to support them manage their long-term health conditions.

Quote 1

Some participants reported that they intentionally changed or stopped the course of their medications when they had no health benefits by taking them. The underlying affective state associated with taking medications was important to mobilize sustained adherence. Participants, who reported not feeling any different by taking medications, seemed to report low motivation to adhere to their prescriptions.

Quote 2

Alternatively, participants who had unpleasant side effects from taking medications reported stopping or skipping their tablets.

Quote 3

The regimens people were prescribed was also mentioned as an important barrier for medication non-adherence. People taking lots of pills reported limited ability to cope with multiple regimens, and those taking few pills reported lack of motivation to continue taking their tablets as prescribed.

Quote 4

Some participants reported beliefs that behaviours can have an accumulative or counteractive effect on each other. In these cases, participants tended to alter their dosages according to the other behaviours enacted at times and/or places related to their mediation taking behaviour. For example, people reported that they intentionally alter or miss their tablets when

For peer review only - http://bmjopen.bmj.com/site/about/guidelines.xhtml

socialising, especially when their social environment did not support them taking their medications.

Quote 5

Some participants also reported difficulties in taking their medicines when they change their everyday routine and/or they are busy. For example, one participant reported forgetting taking his pills when going away for the weekend, whereas another reported forgetting when he has multiple activities to do in a day. Additionally, nurses reported participants' memory issues as a reason for non-intentional non adherence.

Quote 6

Current actions to promote medication adherence and recommendations for an intervention

Participants reported that one way to remember to take their medications as prescribed was to add reminders to their environment. These cues were already associated with activities of their daily routine, and were enacted at close time and space proximity with taking medications.

Quote 7

Patients perceived current practices in primary care as satisfactory in terms of providing them with advice on how to take their medications. Some nurses also reported that they encourage patients to add cues to their environment to help them cope with forgetfulness. Another nurse also reported that she advises patients about how to cope with their underlying affective state during the course of their medications.

Quote 8

However, both patients and nurses reported challenges with the time available within a consultation to address each patient's needs regarding their medications. Moreover, participants reported challenges with their abilities to support themselves (in the case of patients) or provide support to patients (in the case of nurses) to adhere to their prescriptions daily and/or during conflicting conditions.

For peer review only - http://bmjopen.bmj.com/site/about/guidelines.xhtml

Acceptability of the Interactive Voice Response to support reasons for non-adherence to cardio-metabolic medications.

Quote 9

Both nurses and patients expressed favourable views on an automated, telephone based intervention and suggested that such an intervention could be more effective if it was tailored to each patient's reasons for not taking medicines as prescribed.

Quote 10

Acceptability of the IVR delivery mode

IVR seemed to be an acceptable platform to deliver messages for medication adherence for the majority of the participants. Specifically, participants liked the voice delivering the call, the navigation options and the voice recognition software. Even one person, who was reluctant about the automatic element of the call, reported that receiving an automated call would have been better than forgetting medications.

Quote 11

Participants seemed to also like the interactive element of the IVR delivery mode, which they used to report missing some of their dosages and/or to ask for advice on how to cope with non-adherence.

Quote 12

Acceptability of the intervention content

Most participants expressed favourable views about the voice messages with information about the health consequences of adhering or not adhering to their prescriptions, especially since Hypertension and/or T2DM can be asymptomatic. Specifically, participants highlighted the importance of messages that reminded them of the reasons they need to take their medicines as prescribed. Participants reported that receiving the messages at occasions they usually find challenging to adhere to their medications could support them take their tablets as prescribed.

Quote 13

Additionally, most participants liked simple messages that prompted them to take their medications at a due time and facilitated conscious thinking of their behaviour.

For peer review only - http://bmjopen.bmj.com/site/about/guidelines.xhtml

Acceptability of the Interactive Voice Response to support reasons for non-adherence to cardio-metabolic medications.

Quote 14

However, messages that focused on information about the duration of their health condition rather than ways to support them take their medications, and messages asking them to put effort in self-monitoring their medication taking behaviour (e.g. keeping a diary every day) seemed not to be acceptable.

Quote 15

Personalised voice messages (i.e. that included patients' names) seemed to be satisfactory to the majority of participants.

Quote 16

DISCUSSION

Principal findings

Beliefs about the need to take medications, the number of prescribed doses and the ability to cope with the underlying affective state within challenging contexts were mentioned as important reasons for adherence to prescribed medications. Nurses reported time constraints to address each patient's different reasons for medication non adherence, and limited efficacy to support them between and sometimes during the primary care consultations. Both patients and nurses had favourable views on an automated, telephone based intervention and recommended messages to be tailored to each patient's needs for taking medications. Patients expressed positive experiential feedback about the IVR and provided recommendations for intervention content and delivery mode. Specifically, participants liked the voice delivering the messages and the voice recognition software. For intervention content, patients expressed a preference for messages with tailored information about health consequences and simple medication reminders. The interactive element of the interventions seemed to be most useful for people to ask support when they had missed their tablets. Including elements of personalisation would increase uptake and initial engagement with the intervention.

Strengths and limitations

This study invited patients whose practice records suggested that they were not refilling their prescriptions and/or not managing their blood pressure. Insights obtained from these patients

For peer review only - http://bmjopen.bmj.com/site/about/guidelines.xhtml

can usefully inform the content and delivery of a medication adherence intervention within primary care. However, consideration should be given when translating these results to different populations (e.g. patients who do not attend practices or those not participating in research) and other settings (e.g. community).

Comparison with existing literature

Previous studies have investigated the reasons for medication non adherence (21), as well as patients or practitioners' views of an intervention to address non-adherence (22, 23). In accordance with previous research, participants' attitudes about the need to take medications, especially when they are prescribed complex medication regimens (24), seem to be important reason for medication non-adherence. Similarly, previous intervention development and feasibility studies suggested that automated, telephone-based interventions should remind people about their prescription plan, and provide patients with the options of interactive communication and tailored intervention content (25, 26). However, none of these studies had investigated the underlying determinants that account for non-adherence in patients with Hypertension and/or T2DM, whether and how current practices address these determinants, or considered multiple views and recommendations on intervention delivery mode and content to support adherence to prescribed medicines. Most importantly, none of the previous studies have investigated the acceptability of the IVR intervention to address the reasons of medication non adherence in the UK primary care setting, which is an important element when developing novel interventions (27).

Implications for research and practice

IVR seems to be an acceptable mode to influence the determinants of medication adherence in patients with Hypertension and/or T2DM. This study suggests that within the time constraints and limited resources of the primary care, practitioners could briefly assess non adherent patients' INA and NINA reasons for not taking their medicines as prescribed and signpost them to an IVR medication adherence intervention. The results of this study suggest that reminders of the behaviour and tailored information about health consequences are acceptable determinants to promote adherence.

Unanswered questions and future research

For peer review only - http://bmjopen.bmj.com/site/about/guidelines.xhtml

Future studies aiming to develop such interventions in primary care could usefully tailor the intervention to each patient's needs by mapping the voice messages to their reasons for adherence and non-adherence to their medicines. Future studies could integrate participants' views on barriers and facilitators to uptake, retention and engagement with such interventions. Participants' views and recommendations on the delivery mode, such as the clarity, tone, volume, intensity, speed rate, rhythm, and vocal expression of the messages, the navigation options and the frequency and duration of the calls should be further explored. Additionally, future studies could explore participants' views, satisfaction, mastery and actions upon the content of the voice messages, and obtain recommendations for improvement. Future studies could also explore whether these or additional components impact on medication adherence (28), using rigorous designs.

Authors' contributions and competing interests

SS and AK designed the study. AK conducted all tasks involved in completing this study, with substantial input from SS. Authors have no competing interests.

Data sharing statement

Documents describing the process and the data of analysis are available by request.

For peer review only

Acceptability of the Interactive Voice Response to support reasons for non-adherence to cardio-metabolic medications.

REFERENCES

1. National Institute of Health and Care Excellence. Medicine adherence. Involving patients in decisions about prescribed medicines and supporting adherence. 2009.
2. World Health Organisation. Adherence to long-term therapies. Evidence for action. 2003.
3. Chowdhury R, Khan H, Heydon E, et al. Adherence to cardiovascular therapy: a meta-analysis of prevalence and clinical consequences. Eur Heart J. 2013;34(38):2940-8.
4. Department of Health. Improving the use of medicines for better outcomes and reduced waste. An action plan. 2012.
5. Bailey JE, Wan JY, Tang J, et al. Antihypertensive medication adherence, ambulatory visits, and risk of stroke and death. J Gen Intern Med. 2010;25(6):495-503.
6. Barber N. Should we consider non-compliance a medical error? Qual Saf Health Care. 2002;11(1):81-4.
7. Marshall IJ, Wolfe CD, McKevitt C. Lay perspectives on hypertension and drug adherence: systematic review of qualitative research. BMJ. 2012;345:e3953.
8. Nieuwlaat R, Wilczynski N, Navarro T, et al. Interventions for enhancing medication adherence. Cochrane Database Syst Rev. 2014;11:CD000011.
9. Kassavou A, Sutton S. Supporting medication adherence using Interactive Voice Response (IVR): development and delivery of a theory and evidence based intervention. The European Health Psychologist. 2016;18.
10. Tsoli S, Sutton S, Kassavou A. Interactive Voice Response interventions targeting behaviour change: A systematic literature review with meta-analysis and meta-regression. Submitted for publication.
11. Kassavou A, Sutton S. Automated telecommunication interventions to promote adherence to cardio-metabolic medications: meta-analysis of effectiveness and meta-regression of behaviour change techniques (under review).
12. Statistica. Number of mobile voice call minutes in the United Kingdom (UK) from 2007 to 2015 (in billion minutes). 2016.
13. Statistica. Number of fixed voice call minutes in the United Kingdom (UK) from 2007 to 2015 (in billion minutes). 2016.

For peer review only - http://bmjopen.bmj.com/site/about/guidelines.xhtml

14. Smith DH, O'Keeffe-Rosetti M, Owen-Smith AA, et al. Improving Adherence to Cardiovascular Therapies: An Economic Evaluation of a Randomized Pragmatic Trial. Value Health. 2016;19(2):176-84.

15. Chamberlain K, Camin P, Yardley L. Qualitative analysis of experience: grounded theory and case studies. In: Marks D, Yardley L, (eds.) Research Methods for Clinical and Health Psychology. London: SAGE publications; 2004.

16. Farmer A, Hardeman W, Hughes D, et al. An explanatory randomised controlled trial of a nurse-led, consultation-based intervention to support patients with adherence to taking glucose lowering medication for type 2 diabetes. BMC Fam Pract. 2012;13:30.

17. Sutton S. Stage theories. In: Norman MCP, (ed.) Predicting and changing health behaviour: Research and practice with social cognition models. 9. Buckingham: Open University Press; 2015.

18. Kassavou A, French D, Chamberlain K. How do environmental factors influence walking in groups. Journal of Health Psychology. 2015;20(10):1328-1339.

19. Kendall M, Murray SA, Carduff E, Worth A, Harris F, Lloyd A, et al. Use of multiperspective qualitative interviews to understand patients' and carers' beliefs, experiences, and needs. BMJ. 2009;339:b4122.

20. Kassavou A, Turner A, French DP. The role of walkers' needs and expectations in supporting maintenance of attendance at walking groups: a longitudinal multi-perspective study of walkers and walk group leaders. PLoS One. 2015;10(3):e0118754.

21. De Simoni A, Mant J, Sutton S. Adherence to medication in stroke survivors dependent on caregivers. British J Gen Pract. 2015;65(640):e789-91.

22. Lecouturier J, Cunningham B, Campbell D, et al. Medication compliance aids: a qualitative study of users' views. British J Gen Pract. 2011;61(583):93-100.

23. Nolan T, Dack C, Pal K, et al. Patient reactions to a web-based cardiovascular risk calculator in type 2 diabetes: a qualitative study in primary care. British J Gen Pract. 2015;65(632):e152-60.

24. Jamison J, Graffy J, Mullis R, et al. Stroke sursivors', caregivers' and GPs' attitudes towards a polypill for the secondary prevention of stroke: a qualitative interview study. BMJ Open. 2016; 6:e010458 doi:10.1136/bmjopen-2015-010458

For peer review only - http://bmjopen.bmj.com/site/about/guidelines.xhtml

Acceptability of the Interactive Voice Response to support reasons for non-adherence to cardio-metabolic medications.

25. Rana Y, Haberer J, Huang H, et al. Short message service (SMS)-based intervention to improve treatment adherence among HIV-positive youth in Uganda: focus group findings. PLoS One 2015;10(4):e0125187.
26. Osborn CY, Mulvaney SA. Development and feasibility of a text messaging and interactive voice response intervention for low-income, diverse adults with type 2 diabetes mellitus. J Diabetes Sci Technol. 2013;7(3):612-22.
27. Craig P, Dieppe P, Macintyre S, et al. Developing and evaluating complex interventions: the new Medical Research Council guidance. BMJ. 2008;50(5):587-92
28. Jackson C, Eliasson L, Barber N, Weinman J. Applying COM-B to medication adherence. A suggested framework for research and interventions. The European Health Psychologist. 2014; 16(1).

For peer review only

For peer review only - http://bmjopen.bmj.com/site/about/guidelines.xhtml

Reasons for non-adherence to medication and acceptability of an interactive voice recognition intervention in patients with hypertension and type 2 diabetes within the primary care. A qualitative study

No	Quote
1	"*I think probably they don't think they need them to be honest with you. 'I don't need them what are you giving me these for' yeah 'I'm well look at me, I've been like this all me life*" patient B2<br>"*the trouble with diabetes is people don't realise that they've got it and they go on for a couple of years before it's discovered and they probably have gone all this time and not felt ill at all and then they've been given a tablet so they haven't really had a time when they felt ill*" patient B4<br>"*I suppose, deep down are not really happy to be taking medication and they think if they ignore the problem then, or they keep the tablets on the side and they think 'oh I'll take it if I really need it*' nurse A5
2	"*He say that he take one tablet one day, two tablets, second tablet second day and it help but after for example after second tablet on the fourth day it don't help any more ....[he became] accustomed with this tablet with the medications [and don't want to take more]. He went more times to the GP and they gave the same. The tablets did nothing*" patient B17<br>"*I don't really ever consider that I've got high blood pressure. I just don't think about it… with blood pressure it's just how I feel I don't know what it feels like. It is, like you say there's nothing really to tell me day to day* " patient B14<br>"*I often think all these six going down my throat all in one go and they're all releasing their selves but I don't feel any different no...You don't realise what these tiny little things have got in them do you?"* patient B4<br>"*If they're feeling well they'll drop it out or, drop down … they think they don't need it .. They just don't want to take something every day*" nurse A4
3	"*the side effects there were which made me feel ill, there's no point in taking pills that make you feel ill is there?*" patient B2<br>"*I felt as though I was walking through treacle, I wasn't right at all so no we don't take that one anymore*" patient B10<br>"*I'll come across people who won't take something the doctor has prescribed because they're getting side effects that they don't like. For example Metformin which gives them tummy ache and the doctor might have prescribed say two or three doses a day and they'll only take one...generally speaking Metformin in the first couple of weeks can cause a lot of indigestion, nausea, tummy aches that kind of thing and often patients don't like it, so they stop taking it*" nurse A5
4	"*If I'm routinely asked to take a pill without really knowing why I'm taking it, it's not a very good incentive to take that pill…. they may walk out of this surgery thinking 'do I really need to take this [medication]?', lose the prescription or not quite make it to the chemist*" patient B13<br>"*Sometimes I think it's that they, they've got so much medication, they say 'I've got so much*

For peer review only - http://bmjopen.bmj.com/site/about/guidelines.xhtml

	medication I forget'" nurse A3
5	"*The only time I don't take it, if I go to a birthday party or wedding or whatever where you abuse yourself with a bit too much alcohol, I think I'd rather not take the tablets because I don't want it interfering with the alcohol in me, there's enough poison in there as it is"* patient B11<br>*"Yeah, I won't take it if I'm out, if we go out for dinner I won't take my tablets because I don't want other people to see me taking them*" patient B18
6	"*I just take them when I get up and if I get up in a rush then that is when I can forget. I will take the cardinipine later in the day but if I've remembered I've forgotten after about lunchtime*" patient B8<br>"*Oh well I got down to me daughter's in Kent once, when was that? Not last Christmas, the Christmas before and I'd forgotten, I'd taken most of me pills but I'd forgotten half of them so we had to go round to the local doctors and get a prescription, luckily I'd got a copy of my prescription what I take*" patient B1<br>"*memory problems, so we would see maybe forgetfulness, living on their own nobody to prompt them*" nurse A1<br>"*I came across a patient the other day who admitted that he took it when he remembered and that was possibly two, three times a week*" nurse A5
7	"*[In the morning] I have a coffee I put the mug of milk [for the coffee] in the microwave…before I go to bed I put the pills in the microwave…to make me remember and get into the habit of it, you don't have your coffee until you've had your medication otherwise you don't have a coffee. You must associate that and put them together, I do and it works*" patient B3<br>"*I suppose it's quite a little routine isn't it? I actually put a handkerchief by the bed so that I wake up either when the alarm goes or usually I wake up half an hour or so before the alarm and then doze and I find it and take it with a shot of water, I have a bottle of water by the bed*" patient B5<br>"*I think it's about patterns in my life so every morning I have a tray, that's where my phone, my keys are, everything I need for the next day I put on that tray ... so every morning I'd put that [tablet] on my little tray, so really just finding somewhere every day where I've got the things that I need for the next day has been a help to me. Because when I did not have that pattern I'd go to work and 'oh I need my pill now, it's at home'*" patient B14
8	"*Just a lot of encouragement, there was a lot of contact and checking 'have you done this' and 'did you do that' or 'well done you', trying to give them a bit of a boost. I think that's about the best you can do*" patient B10<br>"*I said to her 'would you go out the house without cleaning your teeth', 'no', 'so put it by your toothbrush*" nurse A1<br>"*The other thing I tell them is that how you think you feel is often not a good indication of*

For peer review only - http://bmjopen.bmj.com/site/about/guidelines.xhtml

	actually what state you are in because you just get used to feeling in a certain way" nurse A3
9	"*I think if you've been advised to take it and you don't what can they do? I don't see what help they can do*" patient B6<br>*"I had a new patient's appointment on Tuesday afternoon and she's got a set list of things that she's got to do, she's got to go through what medication you're on, she's got to test your urine, she's got to take your blood pressure and ask these questions and she's got 10 minutes to do it*" patient B9<br>"*Yeah on the prescriptions it will say "take one tablet daily or every day"... people don't want to sit and read it*" nurse A1<br>"*They don't actually understand how the stuff works in the first place so but, but I know that I've had patients back who are saying 'I didn't know that' and I know that I told them it two years ago because it's documented in their notes so they don't always listen either that's the other thing… But we really haven't got time to be going into everybody's medications and why and how they take them.. I just think you can't go round people's houses and ram their pills down their throat twice a day can you*" nurse A4<br>"*Sometimes patients say things to you and then you find out when you actually check their medications or prescriptions that they're not doing what you asked them to do really .. and I suppose there's always this feeling of 'I'll do something about it' very motivated, leave the surgery, 'I'll sort it out, not going to have medicines I'm going to sort this problem out' but actually when it comes to it, it's very difficult to change some lifestyle*" nurse A5
10	"*If it was set automatically that would be brilliant… as long as it was automatic and didn't cost any money; for the health service, not just me*" patients B8<br>"*It's like there's not a pill that will sort everyone out. It's a very complicated thing you really need to fit the answer to the person so you need to find out from them… in order to get someone to take their medication you've got to understand how they fit their world together…so if someone doesn't establish routines you've got to find out how they're keeping their life together*" patient B5<br>"*One size doesn't fit all really does it? Everybody's different and what works for one person doesn't always work for another… ask them what they would prefer I think that could be good*" patient B9<br>"*I think it would need to be different for different people I don't think you could do that across the board*" nurse A5
11	"*Now that you've told me it's not a person I'm talking to I can say what I like can't I?..A lot of people when they pick up that phone are going to think they are talking to a real person. They're not. It's a nice, it's well pitched, the lady is a pleasant voiced lady, it doesn't sound like a cold call or anything…[voice recognition] it's better than pressing buttons, I hate pressing*

For peer review only - http://bmjopen.bmj.com/site/about/guidelines.xhtml

	buttons it's awful" patient B13<br>"*I don't know how to explain it but to be actual talk to a person than a machine is entirely different … that might put certain people off but on the other hand it might think 'thank goodness that came through I'll take my medication'*" patient B6<br>"*So many firms if you press this and you press that, press one, press two, press three. I: Would you prefer that or would you prefer to speak to a (?) R: Yeah this is better, that's much better*" patient B8
12	"*If you've got something wrong with you then you probably wait a few months to go to the doctors…or they'll think 'oh I can get over this' and so on but really in the background in their mind they are slightly worried and they might want a bit more information. I: So do you think they would do that through that system? R: I think they would as a starting point because it's not going, it's not talking on a one to one with somebody, you're actually just accessing an accurate database which is reliable* " patient B16<br>"*I did forget to take my tablets this morning, can I still take them now or should I just wait for the next day?*" patient B18 inbound call<br>"*if I ring up now and say 'oh my prescription's run out can I order a new prescription?'*" patient B12
13	"*R: people get it into their heads that they feel alright and therefore they don't need to take it. I thought it was good in the message that you said that they need to take it regularly in order to maintain, to keep their blood pressure down.*<br>*I: do you think this information is not known to people?*<br>*R: maybe they choose to ignore it. They probably do but high blood pressure is one of those things that unless you actually feel it you're not aware that it's a problem*" patient B9
14	"*Hello Mrs, have you taken your drugs today" just to remind them*" patient B2<br>"*I'm thinking if they ring up, it's like some of my medication like the warfarin I have to take it at a certain time if I don't take it then I've basically got to wait 24 hours and take the next lot. If it says 'have you taken your medication?' you say 'no' then that comes across and say 'oh best I'd take it'*" patient B12<br>"*I got a call at midday saying 'have you taken your medications today?'…it would cause me to think before I answered [laughs]*" patient B16
15	"*It's saying to me, you've been taking this medication for seven years. I don't need them to tell me why, like I know really I just need reminding to take it. The other thing is it says about writing down in your diary but if I was getting the calls I would be like 'what are you talking about?' do you know what I mean? I wouldn't want to be told to write it down. I think if I was that organised that I would write down in a diary I wouldn't need somebody to ring me. I literally would just want it to say 'take your medicine'*" patient B18

For peer review only - http://bmjopen.bmj.com/site/about/guidelines.xhtml

16	"*I think they will because it says your name so you're waiting for it*" patient B18<br>"*It feels like somebody who's closer to you, somebody's who's on, familiar with you so in that respect it's better*" patient B9

For peer review only

For peer review only - http://bmjopen.bmj.com/site/about/guidelines.xhtml

DRAFT interview schedule for pLTCs: "Supporting medication adherence in people with long-term conditions"

FACE-TO-FACE INTERVIEWS WITH PEOPLE WITH LONG TERM CONDITIONS

For peer review only
DRAFT interview schedule pLTCs(v4)

Introduction

- introduce myself
- give an overview of the aims of the interview
- consent issues
    - ask if participants have read the information sheet and if they have any questions
        - if *not*, overview the information included
    - clarify that they can withdraw from the interview at any time, without giving a reason
    - clarify that the interview will be recorded, explain the use of transcripts and the confidentiality issues
- obtain signed consent

For peer review only - http://bmjopen.bmj.com/site/about/guidelines.xhtml

DRAFT interview schedule for pLTCs: "Supporting medication adherence in people with long-term conditions"

First interview

Assessment of the factors that influence medication adherence and current medication taking

- How many medications have you been prescribed?
- Could you describe to me what your medications are?
- What is/are the reason(s) you take your medications?
- What do you expect by taking your medications?
- How do you take your medications?
- When do you take your medications?
- Where do you usually take your medications?
- What do you usually do when you take your medications?
- What helps you to take your medications regularly? / What do you do to help you take your medications?
- Do you take all these medications? / How many of these medications do you take?
    - If yes:
        - what helps you take all your medications as prescribed?
    - If not:
        - which of these do you take and which you do not take?
        - what might be the reasons?
        - what do you think would make it easier to take all your medications as prescribed?
        - what could help you address these reasons?
        - how do you think this could be done?

For peer review only - http://bmjopen.bmj.com/site/about/guidelines.xhtml
For peer review only
DRAFT Interview schedule pLTCs(v4)

DRAFT interview schedule for pLTCs: "Supporting medication adherence in people with long-term conditions"

- what kind of information/support would you need? / could you give me an example?
- do you think a text or voice message intervention could help you to achieve this?

Debriefing questions

- Is there anything else you would like to add about medication adherence?
- Are there any questions about the procedure generally?

Interview prompts

Identifying thoughts/ views:

- Could you please tell me a bit more about that?
- Could you please give me an example of ....?
- Anything else?

Identifying meanings:

- Please correct me if I' m wrong, you said that (rephrase what the interviewee said)...do you have any ideas how that might work?
- Why do you think this is important to you/ to people with long term conditions?
- What do you mean by....?
- What does this depends on?

For peer review only - http://bmjopen.bmj.com/site/about/guidelines.xhtml

DRAFT interview schedule for pLTCs: "Supporting medication adherence in people with long-term conditions"

Second interview

Instructions to participants

We will shortly be beginning a study to support people with long-term conditions to take their medications as prescribed.

For this study, we have developed some intervention materials about people's reasons in taking or not taking their medications. We want to check that people understand the intervention materials in the way that we meant them. To do this, I am going to ask you to 'think-aloud' as you go through these materials. So I want you to tell me everything that you are thinking as you read/hear all the information and decide how to answer it. I would like you to talk constantly. I do not want you to plan out what you say or try to explain to me what you are saying. Just act as if you are alone in the room. If you are silent for any long period of time, I will ask you to 'keep talking' or I will prompt you with some questions.

For peer review only
DRAFT interview schedule pLTCs(v4)

For peer review only - http://bmjopen.bmj.com/site/about/guidelines.xhtml

Draft interview schedule for HCPs: "Supporting medication adherence in people with long-term conditions"

FACE-TO-FACE INTERVIEWS WITH HEALTH CARE PROVIDERS

Introduction

- introduce myself
- give an overview of the aims of the interview
- consent issues
    - ask if participants have read the information sheet and if they have any questions
        - if *not*, overview the information included
    - clarify that they can withdraw from the interview at any time, without giving a reason
    - clarify that the interview will be recorded, explain the use of transcripts and the confidentiality issues
- obtain signed consent

DRAFT interview schedule HCPs (v5)
For peer review only

For peer review only - http://bmjopen.bmj.com/site/about/guidelines.xhtml

Draft interview schedule for HCPs: "Supporting medication adherence in people with long-term conditions"

Experience with medication adherence

- How many patients have you seen with long term conditions?
- How often do you discuss medication adherence with patients with long term conditions? / How many years have you been addressing mediation adherence?
- How many patients have you seen with diabetes, hypertension or both?
- How often do you discuss medication adherence with patients with diabetes and/or hypertension?

Views on medication adherence and factors that influence medication adherence

- Do people with diabetes and hypertension take all their medications as prescribed? - if no, what percentage of participants do not take all their medications as prescribed?
    - Is there any difference between people with diabetes and people with hypertension? - If yes, how do they differ?/ If no, why do you think so? (*use debriefing questions and prompts*)

Views on factors that influence medication adherence

- What reasons do people with diabetes and/or hypertension report for taking their medications as prescribed?
- What benefits do people with diabetes and hypertension report that they gain from taking their medications as prescribed?
- What people expect from taking their medications?
- What sort of things do people ask you about taking their medications?

For peer review only - http://bmjopen.bmj.com/site/about/guidelines.xhtml
For peer review only
DRAFT interview schedule HCPS (v5)

Draft interview schedule for HCPs: "Supporting medication adherence in people with long-term conditions"

- What is your view about how people with diabetes and/or hypertension take their medications?
- What reasons do people report for not taking all their medications as prescribed?

Current practices to address medication adherence and recommendations for improvement

- What do you usually say to motivate people with diabetes and/or hypertension to take their medications regularly as prescribed?
- What do you usually say to people with diabetes and/or hypertension to support them to take their medications as prescribed?
- What do you usually do to support people with diabetes and/or hypertension to take their medications as prescribed?
- How do you see these practices? how do they support people to take their medications as prescribed?
- When do you think people with long term conditions need support more? - how this could be done?
- Do you think a text or voice message intervention could be helpful to support people with diabetes/hypertension to take their medications ? - if yes, how? if no, why?
- What kind of support do you think an text or voice message intervention could provide?/ What kind of information do you think a text or voice message intervention could provide? Can you give me an example?

DRAFT interview schedule HCPs (v5)
For peer review only

For peer review only - http://bmjopen.bmj.com/site/about/guidelines.xhtml

Draft interview schedule for HCPs: "Supporting medication adherence in people with long-term conditions"

- How often do you think a intervention message could be helpful for people with diabetes and hypertension?
- Do you think a text or voice message intervention could be usefully integrated in the primary care?/ if yes, how do you think such intervention could be integrated in the primary care?/ if no, why do you think so? any recommendations for current practices?

For peer review only

DRAFT interview schedule HCPs (v5)

For peer review only - http://bmjopen.bmj.com/site/about/guidelines.xhtml

Draft interview schedule for HCPs: "Supporting medication adherence in people with long-term conditions"

Debriefing questions

- Is there anything else you would like to add about medication adherence?
- Are there any questions about the procedure generally?

Interview prompts

Identifying thoughts/ views:

- Could you please tell me a bit more about that?
- Could you please give me an example of ....?
- Anything else?

Identifying meanings:

- Please correct me if I' m wrong, you said that (rephrase what the interviewee said)...do you have any ideas how that might work?
- Why do you think this is important to you/ to people with long term conditions?
- What do you mean by....?
- What does this depends on?

For peer review only
DRAFT interview schedule HCPs (v5)

For peer review only - http://bmjopen.bmj.com/site/about/guidelines.xhtml

BMJ Open

Reasons for non-adherence to cardio-metabolic medication, and acceptability of an interactive voice response intervention in patients with hypertension and type 2 diabetes in primary care: a qualitative study

Journal:	*BMJ Open*
Manuscript ID	bmjopen-2016-015597.R2
Article Type:	Research
Date Submitted by the Author:	22-Jun-2017
Complete List of Authors:	Kassavou, Katerina; University of Cambridge, Public Health and Primary Care ; University of Cambridge,<br>Sutton, Stephen; University of Cambridge,
**Primary Subject Heading**:	Cardiovascular medicine
Secondary Subject Heading:	Communication, Diabetes and endocrinology, Medical education and training, Patient-centred medicine, Qualitative research
Keywords:	adherence, interactive voice response, Hypertension < CARDIOLOGY, DIABETES & ENDOCRINOLOGY, ORAL MEDICINE

SCHOLARONE™ Manuscripts

For peer review only

Reasons for non-adherence to cardio-metabolic medication, and acceptability of an interactive voice response intervention in patients with hypertension and type 2 diabetes in primary care: a qualitative study

Aikaterini Kassavou and Stephen Sutton

Corresponding author:

Aikaterini Kassavou, BSc in Psychology, MSc in Clinical Health Psychology, PhD in Health Psychology. Research Associate, Behavioural Science Group, University of Cambridge, School of Clinical Medicine, Institute of Public Health and Primary Care, The Primary Care Unit, Forvie Site, Robinson Way, Cambridge, CB2 0SR. kk532@medschl.cam.ac.uk. +44(0)1223330456

Co-author:

Stephen Sutton, BSc in Combined Studies, Psychology main, MSc in Social Psychology, MSc in Computer Science, PhD in Social Psychology. Professor in Behavioural Science, University of Cambridge, Cambridge, UK.

Keywords or phrases: Medication adherence, interactive voice response, hypertension, type 2 diabetes.

Word count: 5,046 (excluding title page, abstract, references, figures and tables).

For peer review only

Reasons for non-adherence to cardio-metabolic medications, and acceptability of the Interactive Voice Response to support these reasons.

ABSTRACT

Objectives: This study explored the reasons for patients' non-adherence to cardio-metabolic medications; and tested the acceptability of the Interactive Voice Response (IVR) as a way to address these reasons, and support patients, between primary care consultations.

Design, method, participants and setting: The study included face-to-face interviews with 19 patients with Hypertension and/or type 2 Diabetes Mellitus, selected from primary care databases, and presumed to be non-adherent. Thirteen of these patients pre-tested elements of the IVR intervention few months later, using a think-aloud protocol. Five practice nurses were interviewed. Data were analysed using multi-perspective, and longitudinal, thematic analysis.

Results: Negative beliefs about taking medications; the complexity of prescribed medication regimens; and the limited ability to cope with the underlying affective state, within challenging contexts; were mentioned as important reasons for non-adherence. Nurses reported time constraints to address each patient's different reasons for non-adherence, and limited efficacy to support patients, between primary care consultations. Patients gave positive experiential feedback about the IVR messages, as a way to support them take their medicines; and provided recommendations for intervention content, and delivery mode. Specifically, they liked the voice delivering the messages, and the voice recognition software. For intervention content, they preferred messages that were tailored, and included messages with "information about health consequences", "action plans", or simple reminders for performing the behaviour.

Conclusions: Patients with Hypertension and/or type 2 Diabetes, and practice nurses, suggested messages tailored to each patient's reasons for non-adherence. Participants recommended IVR as an acceptable platform to support adherence to cardio-metabolic medications, between primary care consultations. Future studies could usefully test the acceptability, and feasibility, of tailored IVR interventions to support medication adherence, as an adjunct to primary care.

For peer review only - http://bmjopen.bmj.com/site/about/guidelines.xhtml

Reasons for non-adherence to cardio-metabolic medications, and acceptability of the Interactive Voice Response to support these reasons.

Strength and limitations of this study

- Participants: patients presumed to be non-adherent to cardio-metabolic medications, and nurses with experience in advising patients for medication taking, recruited by primary care practices within different levels of deprivation.
- Analysis: in-depth, multi-perspective thematic analysis of patients and nurses' views on the reasons for non-adherence; current actions to address these reasons; and the acceptability of IVR delivery mode and content, to support patients' adherence between primary care consultations.
- Design: face-to-face interviews to explore reasons for non-adherence, and think-aloud protocol to explore the acceptability of the IVR.
- Applicability: consideration should be given when translating these results to different populations and settings.

For peer review only

Funding statement. This paper presents independent research funded by the NIHR School for Primary Care Research (Grant Reference Number: 293). The views expressed are those of the author(s) and not necessarily those of the NHS, the NIHR or the Department of Health.

For peer review only - http://bmjopen.bmj.com/site/about/guidelines.xhtml

Reasons for non-adherence to cardio-metabolic medications, and acceptability of the Interactive Voice Response to support these reasons.

INTRODUCTION

Primary care consultations aim to advise and support patients in their decision to take the prescribed medications (1), however a substantial proportion of patients cease or do not take their medications as prescribed, after leaving the consultation room (2,3). Non-adherence to medications contributes to low treatment efficacy and increased NHS costs (4); particularly for people who use health care services regularly, such as patients with either Type 2 Diabetes Mellitus (T2DM) or Hypertension, or those with both health conditions (2,4,5).

Patients' non-adherence is influences by one, or both, intentional (INA) and non-intentional (NINA) reasons (6,7). INA refers to those reasons that are under patients' conscious decisions to miss, or alter, medication regimens; whereas NINA refers to those reasons that are outside patients' conscious consideration of their behaviour. Currently we do not know the reasons for non-adherence, and how to effectively address them (8); and practitioners' time is limited and expensive. Both NICE guidelines (1), and a report commissioned by the Department of Health (4), recommend the development and test of novel interventions to actively involve patients in addressing non-adherence; not only during, but also between primary care consultations.

Interventions using existing telephone systems, are promising platforms to support patients, as an adjunct to primary care (9,10). Our recent systematic review found that automated telephone-based interventions can double the odds to promote medication adherence, when added or compared to usual care (11). Such interventions enable behaviour change using automated, and interactive, voice (IVR) messages. In the UK adults use 216.8 billion minutes in voice calls (142.8 billion via mobile phones and 74 billion minutes via fixed lines) every year (12,13). Despite the major potential to reach a large number of patients, even in remote areas, and facilitate the delivery of low cost medication adherence services (14); the acceptability of such systems has not been tested in the UK primary care setting.

To address this gap, this study aimed to develop, and assess the acceptability, of elements of an IVR intervention to support medication adherence to cardio-metabolic medications. To achieve this, the present study identified the reasons for patients' non-adherence, the current actions to facilitate adherence, and obtained views and

For peer review only - http://bmjopen.bmj.com/site/about/guidelines.xhtml

recommendations about an IVR intervention to influence these reasons, as an adjunct to primary care consultations.

METHODS

Recruitment

The study was approved by the NHS Ethics Committee (REC reference: 14/LO/1872), and the NHS Cambridgeshire and Peterborough Clinical Commissioning Group (CCG), before any participant was approached. Primary care practices within the CCG, with the highest recorded numbers of patients with Hypertension and T2DM; and at different levels of deprivation; were eligible for this study. Sixty-one practices were invited by the research team, and five of them participated in the study; of which four had list sizes smaller than the average, and one larger than the average, when compared to other practices within the CCG. The practices varied in deprivation level. Three practices invited nurses, and four invited patients, to take part in the study.

Nurses eligible for inclusion were those who were involved in advising, encouraging, or supporting patients to adhere to their treatments. Eligible nurses were approached face-to-face by the practice manager, given the study information pack, and invited into the study. Five nurses from three practices provided written informed consent and took part in the interview. Patients eligible for inclusion were those: (a) aged 40 and over; (b) with a primary diagnosis of Hypertension and/or T2DM; (c) who were non-adherent to their medication regimens, as indicated by clinical measures closest to the last refilled medication prescription, or with gaps in ordering or refilling repeat prescriptions during the last three months; and (d) had visited the practice at least once during the last six months.

The practice manager selected eligible patients from the practice database. A practice GP screened the list of eligible patients for the main exclusion criteria (e.g., recent acute health event; diagnosis of dementia; aphasia or other cognitive difficulties; severely impaired hearing or speaking). Practice staff posted study invitation packs to those selected for inclusion. In total 150 invitations were sent to eligible patients, and 19 expressed an interest, and provided written informed consent to participate in the study. We decided to cease recruitment when saturation of the data had been achieved (15). We recruited less nurses than stated in the protocol, because the collected data had reached saturation, after implementing constant comparisons between themes.

For peer review only - http://bmjopen.bmj.com/site/about/guidelines.xhtml

Reasons for non-adherence to cardio-metabolic medications, and acceptability of the Interactive Voice Response to support these reasons.

Interviews

At baseline, face-to-face interviews were conducted using a semi-structured guide to identify the reasons for non-adherence. The interview guide was informed by a review of evidence and theory-based research (16,17). The theoretical concepts that best explained medication adherence change were mapped onto interview questions, and informed the interview schedule (see supplementary files). Interview prompts facilitated the in-depth exploration of meanings, and the identification of themes (18). We asked nurses their views on: (a) patients' experiences with taking medicines, and barriers to adherence; (b) their current actions to address non-adherence; and (c) their recommendations about the development of a telephone-based intervention, as an adjunct to primary care. Interviews with nurses were conducted from January until April 2015. We asked patients about their views on: (a) their condition; (b) medication adherence; (c) barriers and facilitators in taking their medicines; and (d) whether and how an automated, telephone-based intervention could support them take their medications as prescribed. Baseline interviews with patients were conducted from July until September 2015.

Based on the baseline interviews we developed an interactive voice response (IVR) platform to deliver the intervention messages (9). The IVR messages were developed by combining the findings from the baseline interviews, and systematic literature reviews exploring the effective Behaviour Change Techniques (BCTs) to support medication adherence (10,11,19). At the follow up interviews, we used a think-aloud protocol to gain participants' experiential feedback on elements of the IVR. Participants were asked to trigger and complete one, or two IVR calls, and verbalise their thoughts during, and after the calls. Each IVR call lasted approximately 1-5 minutes. Participants were asked and prompted to think aloud their views on the delivery mode and the content of the messages; and asked their recommendations on how IVR messages could be improved, and support them to adhere to their medications best. Follow up interviews with patients were conducted from March until April 2016.

The first author constructed the interview guide, think-aloud protocol, voice messages, and conducted interviews and analysis, with input from the second author. The first author had experience, and training in qualitative research. The second author is also a

For peer review only - http://bmjopen.bmj.com/site/about/guidelines.xhtml

leading expert in the topic of research. Patients were interviewed at their homes (n=11 baseline and n=12 at follow up), at the university (n=7 at baseline), or at their workplace (n=1 at baseline and n=1 at follow up). There was the intention for all the follow-up interviews to take place at participants' homes, to facilitate the collection of contextual information (20). All nurses were interviewed at their practices. All interviews were face-to-face with only the researcher and the interviewee present, apart from one baseline interview, where the interviewee's partner was present to assist with translation. Each baseline interview lasted on average 1 hour, and each follow up interview about 30 minutes. All interviews were digitally recorded and transcribed by an independent service. Transcripts were double-checked against audio recording and field notes, for accuracy by the researcher.

For peer review only

Analysis

We analysed the data collected from the two cross-sectional sets of interviews (i.e., baseline interview with patients and nurses) using in-depth, multi-perspective, thematic analysis (21). All interviews were analysed separately. After that, data were integrated into broader descriptive categories. For baseline interviews, latent themes from patients and nurses' interviews were identified inductively and synthesised into one analysis, capturing both perspectives. Themes from the follow-up interviews were mapped onto those from baseline interviews, and any new themes (e.g. on acceptability of the IVR delivery mode) were treated separately (22).

RESULTS

Participants

In total 37 interviews were conducted: 19 patients were interviewed at baseline, 13 of whom were interviewed few months later, and 5 nurses. Patients' mean age at baseline was 62.3 years, seven of them were female, thirteen reported as primary diagnosis Hypertension, three reported T2DM, and the rest reported diagnosis of co-morbidities of CVD (e.g., stroke) and T2DM. More information about the practices, nurses, and patients' characteristics can be found at Table 1.

For peer review only - http://bmjopen.bmj.com/site/about/guidelines.xhtml

Reasons for non-adherence to cardio-metabolic medications, and acceptability of the Interactive Voice Response to support these reasons.

Table 1: Practice and participants' characteristics.

**Practice** Deprivation level within CCG, area, practice list size on average within CCG, patient/doctor ratio, patient/staff ratio (excluding doctors).	**Participants**					
	**Nurses' pseudonyms and gender**	**Patients**				
		**pseudonyms**	**age**	**gender**	**Primary diagnosis**	**participated at follow up interview***
Average deprived; urban; small; 1136.6/1; 516.6/1	A1 female	B1	80	male	T2DM & hypertension	Yes
		B2	70	male	hypertension (&stroke)	Yes
		B3	67	male	hypertension	yes
		B4	70	female	T2DM	Not interested
		B5	66	male	hypertension	Not interested
		B6	71	female	hypertension	Yes
Least deprived; rural; small; 1208/1; 302/1	A3 female	B7	57	female	hypertension	Moved area
	A4 female	B8	61	female	hypertension	Yes
Less deprived; rural; large; 1275.37/1; 351.8/1		B9	51	female	hypertension	Yes
		B10	64	female	hypertension	Yes
		B11	70	male	hypertension & T2DM	Yes
		B12	64	male	hypertension	Yes
		B13	65	male	hypertension	Yes
		B14	44	male	hypertension	No time
		B15	52	male	hypertension	Yes
		B16	61	male	hypertension and T2DM	Yes
Most deprived; urban; small; 702.6/1; 301/1	A2 female	B17	45	male	hypertension	Excluded
		B18	50	female	T2DM	Yes
Least deprived; rural; small; 1378/1; 393.8/1	A5 female	B19	77	male	T2DM	No time
		Participants mean age in years	62.3			

Average list size CCG N=933,265 of whom n=117,784 patients with hypertension and n=43,296 with diabetes.

For peer review only

For peer review only - http://bmjopen.bmj.com/site/about/guidelines.xhtml

Reasons for non-adherence to cardio-metabolic medications, and acceptability of the Interactive Voice Response to support these reasons.

Note: CCG: clinical commissioning group; A(x) letter code to anonymise nurses (number code to anonymise nurses); B(y) letter code to anonymise patients (number code to anonymise patients); T2DM: Type 2 Diabetes Mellitus; *Yes, if participants took part in the interviews; or reported reasons for not taking part.

Reasons for medication non-adherence

Participants reported that people do not initiate taking their medications as prescribed when they do not understand the need to take medicines, to support them manage their long-term health conditions.

> "*I think probably they don't think they need them to be honest with you. 'I don't need them what are you giving me these for' yeah 'I'm well look at me, I've been like this all me life*" patient B2

> "*the trouble with diabetes is people don't realise that they've got it and they go on for a couple of years before it's discovered and they probably have gone all this time and not felt ill at all and then they've been given a tablet so they haven't really had a time when they felt ill*" patient B4

> "*I suppose, deep down are not really happy to be taking medication and they think if they ignore the problem then, or they keep the tablets on the side and they think 'oh I'll take it if I really need it*' nurse A5

Some participants reported that they intentionally changed or stopped the course of their medications, when they had no health benefits by taking them. The underlying affective state associated with taking medications was important to mobilize sustained adherence. Participants, who reported not feeling any different by taking medications, seemed to report low motivation to adhere to their prescriptions.

> "*He say that he take one tablet one day, two tablets, second tablet second day and it help but after for example after second tablet on the fourth day it don't help any more ....[he became] accustomed with this tablet with the medications [and don't want to take more]. He went more times to the GP and they gave the same. The tablets did nothing*" patient B17

> "*I don't really ever consider that I've got high blood pressure. I just don't think about it… with blood pressure it's just how I feel I don't know what it feels like. It is, like you say there's nothing really to tell me day to day* " patient B14

For peer review only - http://bmjopen.bmj.com/site/about/guidelines.xhtml

Reasons for non-adherence to cardio-metabolic medications, and acceptability of the Interactive Voice Response to support these reasons.

> "*I often think all these six going down my throat all in one go and they're all releasing their selves but I don't feel any different no...You don't realise what these tiny little things have got in them do you?"* patient B4

> "*If they're feeling well they'll drop it out or, drop down ... they think they don't need it .. They just don't want to take something every day*" nurse A4

Alternatively, participants who had unpleasant side effects from taking medications reported stopping or skipping their tablets.

> "*the side effects there were which made me feel ill, there's no point in taking pills that make you feel ill is there?*" patient B2

> "*I felt as though I was walking through treacle, I wasn't right at all so no we don't take that one anymore*" patient B10

> "*I'll come across people who won't take something the doctor has prescribed because they're getting side effects that they don't like. For example, Metformin which gives them tummy ache and the doctor might have prescribed say two or three doses a day and they'll only take one...generally speaking Metformin in the first couple of weeks can cause a lot of indigestion, nausea, tummy aches that kind of thing and often patients don't like it, so they stop taking it*" nurse A5

The regimens people were prescribed was also mentioned as an important barrier for medication non-adherence. People taking lots of pills reported limited ability to cope with multiple regimens, and those taking few pills reported lack of motivation to continue taking their tablets as prescribed.

> "*Sometimes I think it's that they, they've got so much medication, they say 'I've got so much medication I forget'*" nurse A3

> "*If I'm routinely asked to take a pill without really knowing why I'm taking it, it's not a very good incentive to take that pill…. they may walk out of this surgery thinking 'do I really need to take this [medication]?', lose the prescription or not quite make it to the chemist*" patient B13

Some participants reported beliefs that behaviours can have an accumulative or counteractive effect on each other. In these cases, participants tended to alter their dosages according to the

For peer review only - http://bmjopen.bmj.com/site/about/guidelines.xhtml

other behaviours enacted at times and/or places related to their mediation taking behaviour. For example, people reported that they intentionally alter or miss their tablets when socialising, especially when their social environment did not support them taking their medications.

> "*The only time I don't take it, if I go to a birthday party or wedding or whatever where you abuse yourself with a bit too much alcohol, I think I'd rather not take the tablets because I don't want it interfering with the alcohol in me, there's enough poison in there as it is"* patient B11

> *"Yeah, I won't take it if I'm out, if we go out for dinner I won't take my tablets because I don't want other people to see me taking them*" patient B18

Some participants also reported difficulties in taking their medicines when they change their everyday routine and/or they are busy. For example, one participant reported forgetting taking his pills when going away for the weekend, whereas another reported forgetting when he has multiple activities to do in a day. Additionally, nurses reported participants' memory issues as a reason for non-intentional non-adherence.

> "*I just take them when I get up and if I get up in a rush then that is when I can forget. I will take the cardinipine later in the day but if I've remembered I've forgotten after about lunchtime*" patient B8

> "*Oh well I got down to me daughter's in Kent once, when was that? Not last Christmas, the Christmas before and I'd forgotten, I'd taken most of me pills but I'd forgotten half of them so we had to go round to the local doctors and get a prescription, luckily I'd got a copy of my prescription what I take*" patient B1

> "*memory problems, so we would see maybe forgetfulness, living on their own nobody to prompt them*" nurse A1

> "*I came across a patient the other day who admitted that he took it when he remembered and that was possibly two, three times a week*" nurse A5

Current actions to promote medication adherence, and recommendations for an intervention

For peer review only - http://bmjopen.bmj.com/site/about/guidelines.xhtml

Reasons for non-adherence to cardio-metabolic medications, and acceptability of the Interactive Voice Response to support these reasons.

Participants reported that one way to remember to take their medications as prescribed was to add reminders to their environment. These cues were already associated with activities of their daily routine, and were enacted at close time and space proximity with taking medications.

> *[In the morning] I have a coffee I put the mug of milk [for the coffee] in the microwave…before I go to bed I put the pills in the microwave…to make me remember and get into the habit of it, you don't have your coffee until you've had your medication otherwise you don't have a coffee. You must associate that and put them together, I do and it works*" patient B3

> "*I suppose it's quite a little routine isn't it? I actually put a handkerchief by the bed so that I wake up either when the alarm goes or usually I wake up half an hour or so before the alarm and then doze and I find it and take it with a shot of water, I have a bottle of water by the bed*" patient B5

> "*I think it's about patterns in my life so every morning I have a tray, that's where my phone, my keys are, everything I need for the next day I put on that tray ... so every morning I'd put that [tablet] on my little tray, so really just finding somewhere every day where I've got the things that I need for the next day has been a help to me. Because when I did not have that pattern I'd go to work and 'oh I need my pill now, it's at home'*" patient B14

Patients perceived current practices in primary care as satisfactory in terms of providing them with advice on how to take their medications. Some nurses also reported that they encourage patients to add cues to their environment to help them cope with forgetfulness. Another nurse also reported that she advises patients about how to cope with their underlying affective state during the course of their medications.

> "*Just a lot of encouragement, there was a lot of contact and checking 'have you done this' and 'did you do that' or 'well done you', trying to give them a bit of a boost. I think that's about the best you can do*" patient B10

> "*I said to her 'would you go out the house without cleaning your teeth', 'no', 'so put it by your toothbrush*" nurse A1

> "*The other thing I tell them is that how you think you feel is often not a good indication of actually what state you are in because you just get used to feeling in a certain way*" nurse A3

For peer review only - http://bmjopen.bmj.com/site/about/guidelines.xhtml

Reasons for non-adherence to cardio-metabolic medications, and acceptability of the Interactive Voice Response to support these reasons.

However, both patients and nurses reported challenges with the time available within a consultation to address each patient's needs regarding their medications. Moreover, participants reported challenges with their abilities to support themselves (in the case of patients), or provide support to patients (in the case of nurses), to adhere to prescriptions daily, and/or during conflicting conditions.

> "*I think if you've been advised to take it and you don't what can they do? I don't see what help they can do*" patient B6

> *"I had a new patient's appointment on Tuesday afternoon and she's got a set list of things that she's got to do, she's got to go through what medication you're on, she's got to test your urine, she's got to take your blood pressure and ask these questions and she's got 10 minutes to do it*" patient B9

> "*Yeah on the prescriptions it will say "take one tablet daily or every day"... people don't want to sit and read it*" nurse A1

> "*They don't actually understand how the stuff works in the first place so but, but I know that I've had patients back who are saying 'I didn't know that' and I know that I told them it two years ago because it's documented in their notes so they don't always listen either that's the other thing… But we really haven't got time to be going into everybody's medications and why and how they take them.. I just think you can't go round people's houses and ram their pills down their throat twice a day can you*" nurse A4

> "*Sometimes patients say things to you and then you find out when you actually check their medications or prescriptions that they're not doing what you asked them to do really .. and I suppose there's always this feeling of 'I'll do something about it' very motivated, leave the surgery, 'I'll sort it out, not going to have medicines I'm going to sort this problem out' but actually when it comes to it, it's very difficult to change some lifestyle*" nurse A5

Both nurses and patients expressed favourable views on an automated, telephone based intervention; and suggested that such an intervention could be more effective, if it was tailored to each patient's reasons for not taking medicines as prescribed.

> "*If it was set automatically that would be brilliant… as long as it was automatic and didn't cost any money; for the health service, not just me*" patients B8

> "*It's like there's not a pill that will sort everyone out. It's a very complicated thing you really need to fit the answer to the person so you need to find out from them… in order to get*

For peer review only - http://bmjopen.bmj.com/site/about/guidelines.xhtml

> *someone to take their medication you've got to understand how they fit their world together…so if someone doesn't establish routines you've got to find out how they're keeping their life together*" patient B5

> "*One size doesn't fit all really does it? Everybody's different and what works for one person doesn't always work for another… ask them what they would prefer I think that could be good*" patient B9

> "*I think it would need to be different for different people I don't think you could do that across the board*" nurse A5

Acceptability of the IVR delivery mode

IVR seemed to be an acceptable platform to deliver messages for medication adherence for the majority of the participants. Specifically, participants liked the voice delivering the call, the navigation options, and the voice recognition software. Even one person, who was reluctant about the automatic element of the call, reported that receiving an automated call would have been better than forgetting medications.

> "*Now that you've told me it's not a person I'm talking to I can say what I like can't I?..A lot of people when they pick up that phone are going to think they are talking to a real person. They're not. It's a nice, it's well pitched, the lady is a pleasant voiced lady, it doesn't sound like a cold call or anything…[voice recognition] it's better than pressing buttons, I hate pressing buttons it's awful*" patient B13

> "*I don't know how to explain it but to be actual talk to a person than a machine is entirely different … that might put certain people off but on the other hand it might think 'thank goodness that came through I'll take my medication'*" patient B6

> "*So many firms if you press this and you press that, press one, press two, press three. I: Would you prefer that or would you prefer to speak to a (?) R: Yeah this is better, that's much better*" patient B8

Participants seemed to also like the interactive element of the IVR delivery mode, which they used to report missing some of their dosages, and/or to ask for advice on how to cope with non-adherence.

For peer review only - http://bmjopen.bmj.com/site/about/guidelines.xhtml

Reasons for non-adherence to cardio-metabolic medications, and acceptability of the Interactive Voice Response to support these reasons.

"*If you've got something wrong with you then you probably wait a few months to go to the doctors…or they'll think 'oh I can get over this' and so on but really in the background in their mind they are slightly worried and they might want a bit more information. I: So do you think they would do that through that system? R: I think they would as a starting point because it's not going, it's not talking on a one to one with somebody, you're actually just accessing an accurate database which is reliable* " patient B16

"*I did forget to take my tablets this morning, can I still take them now or should I just wait for the next day?*" patient B18 inbound call

"*if I ring up now and say 'oh my prescription's run out can I order a new prescription?*"patient B12

Acceptability of the intervention content

Most participants expressed favourable views about the content of voice messages, which included BCTs, to address reasons for non-adherence. Specifically, they found useful the messages that included "information about the health consequences" of adhering, or not, to their medicines; especially since Hypertension and/or T2DM can be asymptomatic. Specifically, participants highlighted the importance of messages that reminded them of the reasons they need to take their medicines as prescribed. Participants reported that receiving the messages at occasions, which they usually find challenging to adhere to their medications, could support them take their tablets as prescribed.

"*R: people get it into their heads that they feel alright and therefore they don't need to take it. I thought it was good in the message that you said that they need to take it regularly in order to maintain, to keep their blood pressure down.*

I: do you think this information is not known to people?

R: maybe they choose to ignore it. They probably do but high blood pressure is one of those things that unless you actually feel it you're not aware that it's a problem" patient B9

Additionally, most participants liked simple messages that prompted them to take their medications at a due time, and facilitated conscious thinking of their behaviour.

"*Hello Mrs, have you taken your drugs today" just to remind them*" patient B2

For peer review only - http://bmjopen.bmj.com/site/about/guidelines.xhtml

Reasons for non-adherence to cardio-metabolic medications, and acceptability of the Interactive Voice Response to support these reasons.

> "*I'm thinking if they ring up, it's like some of my medication like the warfarin I have to take it at a certain time if I don't take it then I've basically got to wait 24 hours and take the next lot. If it says 'have you taken your medication?' you say 'no' then that comes across and say 'oh best I'd take it'*" patient B12

> "*I got a call at midday saying 'have you taken your medications today?'…it would cause me to think before I answered [laughs]*" patient B16

However, messages that included information about the duration of their health condition, rather than information about how to support themselves adhere to their medications; and messages that asked them to put effort in self-monitoring their medication taking behaviour (e.g. keeping a diary every day); seemed not to be acceptable.

> "*It's saying to me, you've been taking this medication for seven years. I don't need them to tell me why, like I know really I just need reminding to take it. The other thing is it says about writing down in your diary but if I was getting the calls I would be like 'what are you talking about?' do you know what I mean? I wouldn't want to be told to write it down. I think if I was that organised that I would write down in a diary I wouldn't need somebody to ring me. I literally would just want it to say 'take your medicine'*" patient B18

Personalised voice messages (i.e., that included patients' names) seemed to be acceptable to the majority of the participants.

> "*I think they will because it says your name so you're waiting for it*" patient B18

> "*It feels like somebody who's closer to you, somebody's who's on, familiar with you so in that respect it's better*" patient B9

DISCUSSION

Principal findings

Beliefs about the need to take medications, the number of prescribed doses, and the ability to cope with the underlying affective state, within challenging contexts, were mentioned as important reasons for adherence to cardio-metabolic medications. Nurses reported time constraints to address each patient's different reasons for non-adherence, and limited efficacy to support them between, and sometimes during, the primary care consultations. Both patients

For peer review only - http://bmjopen.bmj.com/site/about/guidelines.xhtml

For peer review only

and nurses had favourable views on an automated, telephone-based intervention, and recommended messages to be tailored to each patient's needs for taking medications. Patients expressed positive experiential feedback about elements of the IVR, and provided recommendations for intervention content and delivery mode. Specifically, participants liked the voice delivering the messages, and the voice recognition software. For intervention content, patients expressed a preference for messages that are tailored to their conditions, include "information about health consequences", or simple reminders of the behaviour. The interactive element of the interventions seemed to be most useful for people to request support, when they had missed their tablets. Including elements of personalisation would increase uptake and initial engagement with the intervention.

Strengths and limitations

This study invited patients whose practice records suggested that they were not refilling their prescriptions and/or not managing their blood pressure. Insights obtained from these patients can usefully inform the content, and delivery of, a medication adherence intervention within primary care. However, consideration should be given when translating these results to different populations (e.g. patients who do not attend practices or those not participating in research) and other settings (e.g. community).

Comparison with existing literature

Previous studies have investigated the reasons for medication non-adherence (23), as well as patients or practitioners' views of an intervention to address non-adherence (24,25). In line with previous research, participants' attitudes about the need to take medications, especially when they are prescribed complex medication regimens (26), seem to be important reason for non-adherence. Similarly, previous intervention development, and feasibility, studies suggested that automated, telephone-based interventions should remind people about their prescription plan; and provide patients with the options of interactive communication, and tailored intervention content (27,28). However, none of these studies had investigated the underlying reasons that influence non-adherence to cardio-metabolic medications (i.e., underlying affective state); whether and how current practices address these reasons; or tested considered multiple views and recommendations on intervention delivery mode, and content, to support adherence. Most importantly, none of the previous studies have investigated the

For peer review only - http://bmjopen.bmj.com/site/about/guidelines.xhtml

acceptability of the IVR to address the reasons of non-adherence in the UK primary care setting, which is an important element when developing novel interventions (29).

Implications for research and practice

IVR seems to be an acceptable mode to influence the determinants of medication non-adherence in patients with Hypertension and/or T2DM. This study suggests that within the time constraints, and limited resources of the primary care, practitioners could briefly assess patients' beliefs about taking medications, ability to cope within challenging conditions, and the complexity of prescriptions; and then signpost them to an IVR adherence intervention. The results of this study also suggest reminders of the behaviour, and "information about health consequences", are acceptable techniques to address reasons for non-adherence.

Unanswered questions and future research

Future studies aiming to develop such interventions in primary care could usefully tailor the intervention to each patient's INA and NINA reasons, by mapping the voice messages to these reasons align with the BCTs to address these reasons, and promote adherence. Future studies could integrate participants' views on barriers and facilitators to uptake, retention, and engagement, with such interventions. Participants' views and recommendations on the delivery mode, such as the clarity, tone, volume, intensity, speed rate, rhythm, and vocal expression of the messages, the navigation options, and the frequency and duration of the calls, should be further explored. Additionally, future studies could explore participants' views, satisfaction, mastery, and actions upon the content of IVR interventions, and obtain recommendations for improvement. Future studies could also explore whether these, or additional components proposed by theories such the COM-B model, could impact on medication adherence (30), using rigorous designs.

Authors' contributions and competing interests

SS and AK designed the study. AK conducted all tasks involved in completing this study, with substantial input from SS. Authors have no competing interests.

Data sharing statement

For peer review only - http://bmjopen.bmj.com/site/about/guidelines.xhtml

Reasons for non-adherence to cardio-metabolic medications, and acceptability of the Interactive Voice Response to support these reasons.

Documents describing the process and the data of analysis are available by request.

References

1. Nunes V. Medicines Adherence : involving patients in decisions about prescribed medicines and supporting adherence Full Guideline January 2009 National Collaborating Centre for Primary. Lon [Internet]. 2009;(January):1–364. Available from: http://www.nice.org.uk/nicemedia/pdf/CG76FullGuideline.pdf

2. De Geest S, Sabaté E. Adherence to long-term therapies: Evidence for action. Vol. 2, European Journal of Cardiovascular Nursing. 2003. p. 323.

3. Chowdhury R, Khan H, Heydon E, Shroufi A, Fahimi S, Moore C, et al. Adherence to cardiovascular therapy: A meta-analysis of prevalence and clinical consequences. Eur Heart J. 2013;34(38):2940–8.

4. Department of Health. Improving the use of medicines for better outcomes and reduced waste. An action plan. 2012.

5. Bailey JE, Wan JY, Tang J, Ghani MA, Cushman WC. Antihypertensive medication adherence, ambulatory visits, and risk of stroke and death. J Gen Intern Med [Internet]. 2010;25(6):495–503. Available from: https://www.scopus.com/inward/record.uri?eid=2-s2.0-77955090186&partnerID=40&md5=da6c70e0b10b69d3deedf7ee916c6165

6. Barber N. Should we consider non-compliance a medical error? Qual Saf Health Care. 2002;11(1):81–4.

7. Marshall IJ, Wolfe CDA, McKevitt C. Lay perspectives on hypertension and drug adherence: systematic review of qualitative research. BMJ [Internet]. 2012;345(jul09 1):e3953–e3953. Available from: http://www.bmj.com/cgi/doi/10.1136/bmj.e3953

8. Nieuwlaat R, Wilczynski N, Navarro T, Hobson N, Jeffery R, Keepanasseril A, Agoritsas T, Mistry N, Iorio A, Jack S, Sivaramalingam B, Iserman E, Mustafa RA, Jedraszewski D, Cotoi C HR, Nieuwlaat R, Wilczynski N, Navarro T, Hobson N, Jeffery R, et al. Interventions for enhancing medication adherence. Cochrane database Syst Rev. 2014;11(11):CD000011.

For peer review only - http://bmjopen.bmj.com/site/about/guidelines.xhtml

Reasons for non-adherence to cardio-metabolic medications, and acceptability of the Interactive Voice Response to support these reasons.

9. Kassavou, A Sutton S. Supporting medication adherence using Interactive Voice Response (IVR): development and delivery of a theory and evidence based intervention. Eur Heal Psychol. 2016;18.

10. Tsoli S (2015). Interactive Voice Response interventions targeting behaviour change: A systematic literature review with meta-analysis and meta-regression.MPhil thesis dissertation, Cambridge University, UK.

11. Kassavou, A Sutton S. Automated telecommunication interventions to promote adherence to cardiovascular medications: meta analysis of effectiveness and meta-regression of behaviour change tehcniques. under Rev Heal Psychol Rev. 2017;

12. Statistica. Number of mobile voice call minutes in the United Kingdom (UK) from 2007 to 2015 (in billion minutes). 2016.

13. Statistica. Number of fixed voice call minutes in the United Kingdom (UK) from 2007 to 2015 (in billion minutes). 2016.

14. Smith DH, O'Keeffe-Rosetti M, Owen-Smith AA, Rand C, Tom J, Vupputuri S, et al. Improving Adherence to Cardiovascular Therapies: An Economic Evaluation of a Randomized Pragmatic Trial. Value Heal. 2016;19(2):176–84.

15. Chamberlain K, Camin P YL. Qualitative analysis of experience: grounded theory and case studies. In: Marks D, Yardley L, editor. Research Methods for Clinical and Health Psychology. London: SAGE publications; 2004.

16. Sutton S. Stage theories. In: Norman M, editor. Predicting and changing health behaviour: Research and practice with social cognition models. Buckingham: Open University Press; 2015.

17. Farmer A, Hardeman W, Hughes D, Prevost AT, Kim Y, Craven A, et al. An explanatory randomised controlled trial of a nurse-led, consultation-based intervention to support patients with adherence to taking glucose lowering medication for type 2 diabetes. BMC Fam Pract [Internet]. 2012;13(1):30. Available from: http://bmcfampract.biomedcentral.com/articles/10.1186/1471-2296-13-30

18. Kassavou, A Sutton S. What factors influence medication adherence in people with long term conditions and how can these inform an intervention to support adherence to

For peer review only - http://bmjopen.bmj.com/site/about/guidelines.xhtml

medications in the primary care setting? a longitudinal qualitative research project: a research protocol. Cambridge; 2014.

19. Kassavou A, Sutton S. Developing and pre-testing a tailored interactive voice response (IVR) intervention to support adherence to anti-hypertensive medications: a research protocol. Cambridge; 2016.

20. Kassavou A, French DP, Chamberlain K. How do environmental factors influence walking in groups? A walk-along study. J Health Psychol [Internet]. 2015;20(10):1328–39. Available from: http://journals.sagepub.com/doi/10.1177/1359105313511839

21. Kendall M, Murray SA, Carduff E, Worth A, Harris F, Lloyd A, et al. Use of multiperspective qualitative interviews to understand patients' and carers' beliefs, experiences, and needs. BMJ [Internet]. 2009;339(oct14 3):b4122–b4122. Available from: http://www.bmj.com/cgi/doi/10.1136/bmj.b4122

22. Kassavou A, Turner A, French DP. The role of walkers' needs and expectations in supporting maintenance of attendance at walking groups: A longitudinal multi-perspective study of walkers and walk group leaders. PLoS One. 2015;10(3).

23. De Simoni A, Mant J, Sutton S. Adherence to medication in stroke survivors dependent on caregivers. Br J Gen Pract. 2015;65(640):e789–91.

24. Lecouturier J, Cunningham B, Campbell D, Copeland R. Medication compliance aids: A qualitative study of users' views. Br J Gen Pract. 2011;61(583):93–100.

25. Nolan T, Dack C, Pal K, Ross J, Stevenson FA, Peacock R, et al. Patient reactions to a web-based cardiovascular risk calculator in type 2 diabetes: A qualitative study in primary care. Br J Gen Pract. 2015;65(632):e152–60.

26. Jamison J, Graffy J, Mullis R, Mant J, Sutton S. Stroke survivors', caregivers' and GPs' attitudes towards a polypill for the secondary prevention of stroke: a qualitative interview study. BMJ Open [Internet]. 2016;6(5):e010458. Available from: http://bmjopen.bmj.com/lookup/doi/10.1136/bmjopen-2015-010458

27. Rana Y, Haberer J, Huang H, Kambugu A, Mukasa B, Thirumurthy H, et al. Short Message Service (SMS)-based intervention to improve treatment adherence among

For peer review only - http://bmjopen.bmj.com/site/about/guidelines.xhtml

HIV-positive youth in Uganda: Focus group findings. PLoS One. 2015;10(4).

28. Osborn CY, Mulvaney SA, C.Y. O. Development and feasibility of a text messaging and interactive voice response intervention for low-income, diverse adults with type 2 diabetes mellitus. J Diabetes Sci Technol [Internet]. 2013;7(3):612–22. Available from: http://ovidsp.ovid.com/ovidweb.cgi?T=JS&PAGE=reference&D=emed11&NEWS=N&AN=23759393

29. Craig P, Dieppe P, Macintyre S, Michie S, Nazareth I, Petticrew M, et al. Developing and evaluating complex interventions: the new Medical Research Council guidance. BMJ [Internet]. 2008;337(sep29 1):a1655–a1655. Available from: http://discovery.ucl.ac.uk/103060/%5Cnhttp://www.bmj.com/cgi/doi/10.1136/bmj.a1655

30. Eliasson L, Barber N, Weinman J. Applying COM-B to medication adherence: a suggested framework for research and interventions. Eur Heal Psychol. 2014;7–17.

For peer review only

For peer review only - http://bmjopen.bmj.com/site/about/guidelines.xhtml

Acceptability of the Interactive Voice Response to support reasons for non-adherence to cardio-metabolic medications.

For peer review only

For peer review only - http://bmjopen.bmj.com/site/about/guidelines.xhtml

DRAFT interview schedule for pLTCs: "Supporting medication adherence in people with long-term conditions"

FACE-TO-FACE INTERVIEWS WITH PEOPLE WITH LONG TERM CONDITIONS

Introduction

- introduce myself
- give an overview of the aims of the interview
- consent issues
    - ask if participants have read the information sheet and if they have any questions
        - if *not*, overview the information included
    - clarify that they can withdraw from the interview at any time, without giving a reason
    - clarify that the interview will be recorded, explain the use of transcripts and the confidentiality issues
- obtain signed consent

DRAFT interview schedule pLTCs(v4)
For peer review only

For peer review only - http://bmjopen.bmj.com/site/about/guidelines.xhtml

DRAFT interview schedule for pLTCs: "Supporting medication adherence in people with long-term conditions"

<u>First interview</u>

Assessment of the factors that influence medication adherence and current medication taking

- How many medications have you been prescribed?
- Could you describe to me what your medications are?
- What is/are the reason(s) you take your medications?
- What do you expect by taking your medications?
- How do you take your medications?
- When do you take your medications?
- Where do you usually take your medications?
- What do you usually do when you take your medications?
- What helps you to take your medications regularly? / What do you do to help you take your medications?
- Do you take all these medications? / How many of these medications do you take?
    - If yes:
        - what helps you take all your medications as prescribed?
    - If not:
        - which of these do you take and which you do not take?
        - what might be the reasons?
        - what do you think would make it easier to take all your medications as prescribed?
        - what could help you address these reasons?
        - how do you think this could be done?

DRAFT Interview schedule pLTCs(v4)
For peer review only

- what kind of information/support would you need? / could you give me an example?
- do you think a text or voice message intervention could help you to achieve this?

Debriefing questions

- Is there anything else you would like to add about medication adherence?
- Are there any questions about the procedure generally?

Interview prompts

Identifying thoughts/ views:

- Could you please tell me a bit more about that?
- Could you please give me an example of ....?
- Anything else?

Identifying meanings:

- Please correct me if I' m wrong, you said that (rephrase what the interviewee said)...do you have any ideas how that might work?
- Why do you think this is important to you/ to people with long term conditions?
- What do you mean by....?
- What does this depends on?

For peer review only - http://bmjopen.bmj.com/site/about/guidelines.xhtml

DRAFT interview schedule for pLTCs: "Supporting medication adherence in people with long-term conditions"

Second interview

Instructions to participants

We will shortly be beginning a study to support people with long-term conditions to take their medications as prescribed.

For this study, we have developed some intervention materials about people's reasons in taking or not taking their medications. We want to check that people understand the intervention materials in the way that we meant them. To do this, I am going to ask you to 'think-aloud' as you go through these materials. So I want you to tell me everything that you are thinking as you read/hear all the information and decide how to answer it. I would like you to talk constantly. I do not want you to plan out what you say or try to explain to me what you are saying. Just act as if you are alone in the room. If you are silent for any long period of time, I will ask you to 'keep talking' or I will prompt you with some questions.

For peer review only
DRAFT interview schedule pLTCs(v4)

For peer review only - http://bmjopen.bmj.com/site/about/guidelines.xhtml

Draft interview schedule for HCPs: "Supporting medication adherence in people with long-term conditions"

FACE-TO-FACE INTERVIEWS WITH HEALTH CARE PROVIDERS

Introduction

- introduce myself
- give an overview of the aims of the interview
- consent issues
    - ask if participants have read the information sheet and if they have any questions
        - if *not*, overview the information included
    - clarify that they can withdraw from the interview at any time, without giving a reason
    - clarify that the interview will be recorded, explain the use of transcripts and the confidentiality issues
- obtain signed consent

For peer review only
DRAFT interview schedule HCPs (v5)

For peer review only - http://bmjopen.bmj.com/site/about/guidelines.xhtml

Draft interview schedule for HCPs: "Supporting medication adherence in people with long-term conditions"

Experience with medication adherence

- How many patients have you seen with long term conditions?
- How often do you discuss medication adherence with patients with long term conditions? / How many years have you been addressing mediation adherence?
- How many patients have you seen with diabetes, hypertension or both?
- How often do you discuss medication adherence with patients with diabetes and/or hypertension?

Views on medication adherence and factors that influence medication adherence

- Do people with diabetes and hypertension take all their medications as prescribed? - if no, what percentage of participants do not take all their medications as prescribed?
    - Is there any difference between people with diabetes and people with hypertension? - If yes, how do they differ?/ If no, why do you think so? (*use debriefing questions and prompts*)

Views on factors that influence medication adherence

- What reasons do people with diabetes and/or hypertension report for taking their medications as prescribed?
- What benefits do people with diabetes and hypertension report that they gain from taking their medications as prescribed?
- What people expect from taking their medications?
- What sort of things do people ask you about taking their medications?

For peer review only
DRAFT interview schedule HCPs (v5)

For peer review only - http://bmjopen.bmj.com/site/about/guidelines.xhtml

Draft interview schedule for HCPs: "Supporting medication adherence in people with long-term conditions"

- What is your view about how people with diabetes and/or hypertension take their medications?
- What reasons do people report for not taking all their medications as prescribed?

Current practices to address medication adherence and recommendations for improvement

- What do you usually say to motivate people with diabetes and/or hypertension to take their medications regularly as prescribed?
- What do you usually say to people with diabetes and/or hypertension to support them to take their medications as prescribed?
- What do you usually do to support people with diabetes and/or hypertension to take their medications as prescribed?
- How do you see these practices? how do they support people to take their medications as prescribed?
- When do you think people with long term conditions need support more? - how this could be done?
- Do you think a text or voice message intervention could be helpful to support people with diabetes/hypertension to take their medications ? - if yes, how? if no, why?
- What kind of support do you think an text or voice message intervention could provide?/ What kind of information do you think a text or voice message intervention could provide? Can you give me an example?

For peer review only - http://bmjopen.bmj.com/site/about/guidelines.xhtml
For peer review only
DRAFT interview schedule HCPs (v5)

Draft interview schedule for HCPs: "Supporting medication adherence in people with long-term conditions"

- How often do you think a intervention message could be helpful for people with diabetes and hypertension?
- Do you think a text or voice message intervention could be usefully integrated in the primary care?/ if yes, how do you think such intervention could be integrated in the primary care?/ if no, why do you think so? any recommendations for current practices?

For peer review only

DRAFT interview schedule HCPs (v5)

For peer review only - http://bmjopen.bmj.com/site/about/guidelines.xhtml

Draft interview schedule for HCPs: "Supporting medication adherence in people with long-term conditions"

Debriefing questions

- Is there anything else you would like to add about medication adherence?
- Are there any questions about the procedure generally?

Interview prompts

Identifying thoughts/ views:

- Could you please tell me a bit more about that?
- Could you please give me an example of ....?
- Anything else?

Identifying meanings:

- Please correct me if I' m wrong, you said that (rephrase what the interviewee said)...do you have any ideas how that might work?
- Why do you think this is important to you/ to people with long term conditions?
- What do you mean by....?
- What does this depends on?

For peer review only

DRAFT interview schedule HCPs (v5)

For peer review only - http://bmjopen.bmj.com/site/about/guidelines.xhtml
